# Application of Behaviour Change Techniques in Promoting Physical Activity Among Adults with Chronic Conditions: An Umbrella Review

**DOI:** 10.3390/bs15111448

**Published:** 2025-10-24

**Authors:** Sanying Peng, Fang Yuan, Hongchang Yang, Meilin Li, Xiaoming Yang

**Affiliations:** 1 Department of Physical Education, Hohai University, Nanjing 210024, China; yanghongchang@hhu.edu.cn (H.Y.); 2311010101@hhu.edu.cn (M.L.); 2 College of International Languages and Cultures, Hohai University, Nanjing 210024, China; yuanf@hhu.edu.cn; 3 Physical Education College, Shanghai University, Shanghai 200444, China; 770721@shu.edu.cn

**Keywords:** behaviour change technique, physical activity, chronic condition, umbrella review

## Abstract

This umbrella review examined the application of behaviour change techniques (BCTs) and their associations with physical activity (PA) outcomes in interventions targeting adults with chronic conditions. A comprehensive search of five databases was conducted up to 20 December 2024, identifying eighteen eligible systematic reviews (including nine meta-analyses), encompassing 468 primary studies and over 57,500 participants. BCTs were coded using the BCT Taxonomy v1, and review quality was assessed using AMSTAR 2. Across the included studies, eleven BCTs were most frequently employed, clustering into four core domains: self-regulation, instruction/information, social or contextual support, and modelling. Among these, four BCTs—goal setting (behaviour), social support (unspecified), instruction on how to perform the behaviour, and graded tasks—were consistently associated with significant increases in PA. Subgroup analysis revealed condition-specific patterns: graded tasks combined with social incentives were most effective for metabolic disorders, instructional techniques for cardiovascular disease, combined instruction and social support for musculoskeletal conditions, goal setting for mixed chronic conditions, and pairing action planning with graded tasks for cancer survivors. These findings advance both theoretical and practical understanding of components associated with successful PA interventions and provide a robust evidence base to inform future program design for chronic disease management.

## 1. Introduction

Noncommunicable diseases (NCDs), including cardiovascular diseases, cancers, chronic respiratory diseases, and diabetes, remain the leading global causes of death and disability, accounting for approximately 73% of all deaths worldwide (World Health Organization, 2023a). Within this broader category, chronic conditions—such as cardiovascular and metabolic diseases, cancer, and musculoskeletal disorders—represent a significant subset characterized by their long duration and need for continuous care and self-management (GBD 2019 Diseases and Injuries Collaborators, 2020; World Health Organization, 2023b). These chronic conditions often share overlapping modifiable risk factors, including physical inactivity, tobacco use, unhealthy diet, and excessive alcohol consumption, which collectively contribute to their onset, progression, and associated health burdens (Beaglehole et al., 2011; GBD 2019 Risk Factors Collaborators, 2020).

Physical inactivity has garnered substantial attention among these risk factors due to its pervasive impact on health outcomes. Physical activity (PA) is widely recognized as a critical component in both the prevention and management of chronic conditions, with established benefits for reducing risks of cardiovascular diseases, type 2 diabetes, certain cancers, and musculoskeletal disorders (Bull et al., 2020; Piercy et al., 2018; Warburton et al., 2006). Moreover, regular PA contributes to better mental health, functional independence, and overall quality of life in individuals with chronic conditions (Anderson & Durstine, 2019; Pedersen & Saltin, 2015). Despite this evidence, adherence to recommended PA levels remains suboptimal among adults with chronic conditions. Previous studies indicate that fewer than half of this population engage in sufficient PA (Alqahtani, 2022; Forechi et al., 2018). These findings highlight the urgent need for the development of effective intervention strategies to promote and maintain PA engagement in this high-risk group.

Various interventions incorporating educational, motivational, and technological approaches have emerged. While such interventions show promise in enhancing PA, their effects are often modest and variable across disease types and individual characteristics (Murray et al., 2017; Rhodes & Dickau, 2012). Against this backdrop, integrating theory-informed strategies, particularly those grounded in behaviour change techniques (BCTs), has gained momentum. BCTs are replicable, observable components of an intervention intended to alter behaviour (Michie et al., 2011). The BCT Taxonomy v1 (BCTTv1) was developed to support consistent application, organizing 93 techniques into 16 conceptual clusters to aid in designing, implementing, and reporting behaviour change interventions (Michie et al., 2013). The taxonomy has become a foundational tool in public health and behavioural sciences, improving the precision and replicability of PA interventions (Carraca et al., 2021; Presseau et al., 2015).

Numerous systematic reviews and meta-analyses have examined the BCT applications in PA interventions targeting specific chronic conditions. For instance, Carraca et al. (2021) evaluated the effects of digital BCT-based interventions in populations with metabolic disorders, while Meade et al. (2019) focused on techniques enhancing adherence among individuals with persistent musculoskeletal pain. Other reviews have addressed PA promotion in patients with cancer (Hailey et al., 2022), cardiovascular diseases (Duff et al., 2017), and multiple or mixed chronic conditions (Lin et al., 2022). These reviews consistently identify goal setting, self-monitoring, instruction, and social support as commonly used BCTs that show potential in improving PA behaviours.

However, despite these contributions, the findings across reviews remain inconclusive. Even within the same condition category, results vary considerably regarding which BCTs are most effective and whether the number or type of techniques moderates outcomes. For example, Marley et al. (2017) reported that only interventions longer than three months produced significant PA benefits. In contrast, Lin et al. (2022) emphasized the value of using more than seven BCTs. Such divergence may reflect BCT coding inconsistencies, intervention design and reporting heterogeneity, and limited methodological rigour across studies.

Moreover, most existing reviews adopt a condition-specific focus and rarely facilitate comparisons across chronic disease groups. This fragmented approach hinders the identification of cross-cutting behavioural strategies and limits the development of broadly applicable, scalable interventions. To date, no comprehensive synthesis has systematically evaluated the application and associations of BCTs with PA outcomes across diverse chronic condition populations.

The present umbrella review provides a structured synthesis of BCT use and effectiveness across published systematic reviews to address this gap. Specifically, it aims to (1) identify the most frequently applied BCTs and those consistently associated with improved PA outcomes among adults with chronic conditions and (2) summarize these findings across distinct chronic condition groups—including metabolic, cardiovascular, musculoskeletal, cancer-related, and mixed/general populations—to inform more targeted, evidence-based PA intervention strategies.

## 2. Methods

This umbrella review adhered to the Joanna Briggs Institute Manual for Evidence Synthesis (JBI) (Aromataris et al., 2020). The study selection process was documented using a PRISMA flowchart (Page et al., 2021), and reporting aligned with the Preferred Reporting Items for Overviews of Reviews (PRIOR) (Pollock et al., 2019). The protocol has been registered in the International Prospective Register of Systematic Reviews platform (PROSPERO; registration number CRD42025630806). The only deviation from the original protocol was the restriction of the study population to adults, while all other methodological aspects remained consistent with the registered version.

### 2.1. Search Strategy

Two authors employed pre-designed search strategies to retrieve literature from five electronic databases: PubMed, Embase, Web of Science, PsycINFO, and the Cochrane Library. The search combined Medical Subject Headings (MeSH) with free-text terms and was structured using Boolean operators. The databases were searched to identify relevant studies from their inception until 20 December 2024. In addition, a recursive manual search of the reference lists of the included studies and pertinent reviews was conducted to identify any potentially eligible records. Detailed search strategies are provided in Supplementary Materials S1.

### 2.2. Eligibility Criteria

#### 2.2.1. Population

Studies were eligible if they included adults (aged 18–65 years) diagnosed with one or more chronic conditions, defined according to the World Health Organization as long-term health conditions that develop slowly and require ongoing management (World Health Organization, 2023b). Eligible conditions included major noncommunicable diseases such as cardiovascular and metabolic diseases, cancer, and other long-term disorders. Participants could be recruited from clinical or rehabilitative settings. Studies focusing on acute illnesses, temporary health states, adolescents, children, or older adults were excluded.

#### 2.2.2. Intervention

Eligible interventions had to focus on promoting PA, enhancing PA adherence, or achieving recommended PA levels. Moreover, the intervention must have explicitly coded and reported the BCTs employed. Studies in which the primary intervention target was weight control, quality of life improvement, smoking cessation, or dietary behaviour—and that did not separately report PA-related outcomes—were excluded.

#### 2.2.3. Comparison

Since this umbrella review examines the PA-promoting effects and the application of BCTs in the intervention groups of systematic reviews, there were no specific eligibility criteria for the comparison or control groups.

#### 2.2.4. Outcomes

Studies were required to report explicit PA outcomes, which could be objectively or subjectively measured, including indicators such as PA adherence, compliance with PA recommendations, or quantified changes in PA behaviour (e.g., steps, frequency, duration, or intensity). The intervention’s effect on PA must be described either quantitatively or qualitatively. Reviews were eligible regardless of whether PA outcomes were reported as primary or secondary measures, provided that explicit PA-related results were presented. As this umbrella review is primarily aimed at synthesizing the application and effectiveness of BCTs within PA interventions, no further harmonization of PA metrics across primary reviews was undertaken. Definitions and measurement methods for PA outcomes were retained as reported, provided they reflected behavioural changes in PA. In addition, eligible studies must have provided a quantitative report of the BCTs used in the PA intervention, with BCT classification performed according to BCTTv1. Studies that only reported aggregated BCT data across multiple behavioural interventions were excluded.

#### 2.2.5. Study Design

The review included published systematic reviews, meta-analyses, and primary studies that included randomized controlled trials (RCTs), non-RCTs, quasi-experimental studies, or single-arm trials. Cohort and observational studies without an active intervention and studies offering only a narrative qualitative synthesis of a single trial were excluded.

#### 2.2.6. Eligibility Assessment and Data Extraction

All retrieved records were imported into EndNote 20, and duplicates were removed. Two authors independently screened the remaining records by title and abstract; any disagreements were resolved by consulting a third author. The full texts of studies meeting the inclusion criteria were then independently assessed by two authors to confirm eligibility. Following the full-text screening, a recursive manual search of the reference lists of the included systematic reviews and relevant articles was conducted to identify additional eligible studies. Discrepancies during the full-text review were resolved through discussion and adjudication by a third author.

Data extraction was performed using a pre-designed Excel spreadsheet. One author independently extracted relevant data from the included reviews, while a second author verified the extracted data’s accuracy and completeness; disagreements were resolved through consensus. Extracted data included author names, publication year, title, number and types of trials included in each review, participant conditions and sample sizes, modes and methods of intervention delivery, primary intervention outcomes, PA intervention effects, details regarding the application of BCTs (including types, mean counts, and frequency of occurrence), the effectiveness of BCTs or their components, and the overall quality of evidence.

### 2.3. Quality Assessment

The methodological quality of the included systematic reviews was evaluated using the Assessment of Multiple Systematic Reviews 2 (AMSTAR 2) tool (Shea et al., 2017), which consists of 16 items. Each review was assigned an overall quality rating (high, moderate, or low) based on the aggregated evaluation across all items, following the recommendations provided by AMSTAR 2. Two authors independently conducted the quality assessments, and any discrepancies were resolved by discussion and consultation with a third author.

### 2.4. Data Analysis and Synthesis

Data from the included systematic reviews and meta-analyses were analyzed using a two-tiered approach. First, studies were categorized by chronic condition type and synthesized using narrative qualitative methods. Second, quantitative data concerning the BCT applications within PA interventions were examined in aggregate and condition-specific subgroups. Given that not all included reviews conducted meta-analyses—and due to substantial heterogeneity in PA outcomes among those that did—a narrative descriptive synthesis was applied to summarize intervention effects.

All reported techniques were coded according to BCTTv1 for the BCT-focused analysis to ensure methodological consistency. Key variables extracted included the mean number of BCTs per review frequency of individual BCTs (with high frequency defined as those in ≥50% of studies). The included reviews reported associations between specific BCTs and PA outcomes, including subgroup or moderation analysis findings. The total number of BCTs was also examined in terms of intervention effectiveness. These data were compiled into a structured matrix linking BCT usage with relevant study characteristics.

To evaluate the extent of overlap among included reviews—a critical issue in overviews of reviews—citation coincidence analysis (CCA) was conducted (Kirvalidze et al., 2023). CCA quantifies the proportion of shared primary studies across systematic reviews and provides a standardized overlap measure, with overlap interpreted as very minor (0–5%), minor (6–10%), moderate (11–15%), or substantial (>15%) (Lau et al., 2024). This metric was used to assess the potential impact of data duplication on the synthesis and to strengthen the validity of the overall conclusions.

Descriptive statistics were employed to summarize BCT usage patterns, frequencies, and associations with intervention outcomes. Due to the non-normal distribution of extracted data and inconsistent reporting across reviews, subgroup comparisons were restricted to descriptive and narrative analyses.

## 3. Results

### 3.1. Study Inclusion

**Figure 1:**
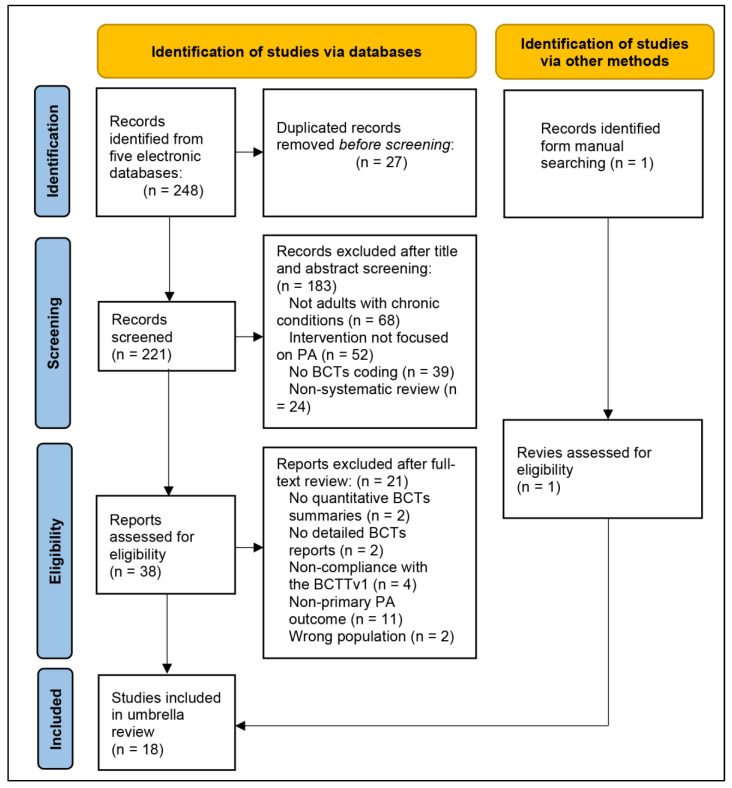
Flowchart of the Literature Screening Process.

The study inclusion process for this umbrella review is presented in Figure 1. Initially, a total of 248 records were identified through electronic database searches. After removing 27 duplicate records, 221 were retained and subjected to title and abstract screening. During this stage, 183 records were excluded primarily because they did not meet the eligibility criteria. Following this stage, 38 records were selected for full-text review. During the full-text review, 21 reports were excluded based on predefined eligibility criteria. Additionally, one record was identified through manual searching, which met the eligibility criteria and was subsequently included in the review. Ultimately, 18 studies (Agirre-Elordui et al., 2024; Ashley et al., 2024; Carraca et al., 2021; Cooper et al., 2023; de Leeuwerk et al., 2022; Duff et al., 2017; Ester et al., 2021; Finne et al., 2018; Grimmett et al., 2019; Hailey et al., 2022; Hallward et al., 2020; Lin et al., 2022; Marley et al., 2017; Mbous et al., 2020; Meade et al., 2019; Meyer-Schwickerath et al., 2022; Willett et al., 2019; X. Zhang et al., 2024) were included in the umbrella review, ensuring that only those meeting the full inclusion standards were considered for analysis.

### 3.2. Quality of the Evidence

The methodological quality of the 18 systematic reviews was assessed using the AMSTAR 2 tool (Shea et al., 2017), which consists of 16 items covering key aspects of systematic review methodology. The reviews were rated as follows: 4 were of high quality (Agirre-Elordui et al., 2024; Carraca et al., 2021; de Leeuwerk et al., 2022; X. Zhang et al., 2024), 9 were of moderate quality (Ashley et al., 2024; Cooper et al., 2023; Duff et al., 2017; Hallward et al., 2020; Lin et al., 2022; Marley et al., 2017; Mbous et al., 2020; Meade et al., 2019; Willett et al., 2019), and 5 were of low quality (Ester et al., 2021; Finne et al., 2018; Grimmett et al., 2019; Marley et al., 2017; Meyer-Schwickerath et al., 2022). All studies consistently adhered to key methodological standards, including articulating clear research questions and inclusion criteria, systematic study selection processes, comprehensive descriptions of included studies, rigorous risk of bias assessments, and transparent disclosure of conflicts of interest. However, areas of concern included the transparency of funding sources, where no study fully met the requirement, and the processes of study selection and data extraction in duplicate, which were not consistently adhered to. Items related to the investigation of publication bias and the explanation of heterogeneity also showed mixed compliance, indicating variability in how these issues were addressed across the reviews. Specifically, nine of the included reviews (Agirre-Elordui et al., 2024; Carraca et al., 2021; de Leeuwerk et al., 2022; Finne et al., 2018; Marley et al., 2017; Mbous et al., 2020; Meyer-Schwickerath et al., 2022; Willett et al., 2019; X. Zhang et al., 2024) conducted publication bias assessments using funnel plots or statistical tests, most of which reported minimal or no evidence of bias. The remaining reviews did not explicitly assess publication bias, which may limit the comparability and transparency of synthesized evidence. These findings highlight strengths in adherence to basic methodological standards but point to areas, such as transparency and bias assessment, that could be improved to enhance the quality and rigour of future systematic reviews in this field. The detailed evaluation is presented in Supplementary Materials S2.

### 3.3. Characteristics of Included Studies

The 18 reviews included in this umbrella review were published between 2016 and 2024, with half performing meta-analyses. The reviews encompassed 468 individual trial studies, most of which were randomized controlled trials (RCTs) (422/468). The remaining studies were non-RCT and quasi-experimental (46/468). All included reviews incorporated RCTs, with 11 reviews (Agirre-Elordui et al., 2024; Ashley et al., 2024; de Leeuwerk et al., 2022; Duff et al., 2017; Finne et al., 2018; Grimmett et al., 2019; Mbous et al., 2020; Meade et al., 2019; Meyer-Schwickerath et al., 2022; Willett et al., 2019; X. Zhang et al., 2024) exclusively including RCT-based studies. The included reviews covered a diverse range of chronic conditions, including metabolic conditions (*n* = 2), cardiovascular conditions (*n* = 2), musculoskeletal conditions (*n* = 3), mixed/general chronic conditions (*n* = 2), and cancer (*n* = 9). Across all studies, there were over 57,543 participants in total.

The PA interventions described in the included studies varied widely. These included digital health interventions, such as those using wearable devices, the internet, mobile messages, and social media, and face-to-face interventions, such as counselling sessions, psychological assessments, and educational information. Many studies utilized a combination of these intervention modes. The delivery of BCTs within these interventions varied accordingly, encompassing structured behavioural counselling, personalized digital feedback, social support features, and self-monitoring tools, each serving as a vehicle for behaviour change implementation. The interventions were primarily delivered in home and community settings. The duration of the interventions varied significantly, ranging from a single contact to interventions lasting up to two years. Only a few reviews reported on follow-up periods, typically 6 months to 2 years, with the most extended follow-up period being 5 years (Grimmett et al., 2019). A comprehensive summary of the characteristics of the included studies is provided in Supplementary Materials S3.

### 3.4. PA Intervention Efficiency and BCT Applications

The umbrella review included nine systematic reviews that conducted quantitative meta-analyses of different PA outcomes and intervention durations. In addition, nine other reviews provided only narrative summaries of intervention effects. Given the high heterogeneity observed in the PA outcomes, intervention durations, and intervention types across the meta-analyses, this review refrained from quantitatively pooling the effect sizes of PA interventions across the included reviews. Across the included reviews, improvements in PA typically referred to increases in objectively or subjectively measured indicators such as steps, duration, frequency, energy expenditure, or adherence to PA recommendations.

All meta-analyses reported significant effects of PA interventions. Most effect sizes were small to moderate, ranging from 0.22 to 0.42. Notably, Carraca et al. (2021) reported a large effect size for face-to-face interventions (effect size = 0.78), while X. Zhang et al. (2024) observed a moderate effect size for the improvement in steps (effect size = 0.56). Marley et al. (2017) summarized the effects of PA interventions across different durations and found significant effects only for interventions lasting more than 3 months. Most studies showed considerable intervention effects in the narrative synthesis of the remaining nine reviews, though variability was observed in the effectiveness of PA interventions during follow-up periods.

Before quantitatively synthesizing the application of BCTs, an overlap analysis was conducted using the CCA (citation coincidence analysis) indicator to assess the overlap of studies in the reviews on chronic conditions within the cancer population. The overlap matrix is presented in Supplementary Materials S4. The analysis revealed a very minimal overlap (CCA = 0.087) among the nine studies included in cancer-related reviews. No overlap was found in the other included systematic reviews’ original studies.

Seventy-two unique BCTs were identified among the PA interventions summarized across the included reviews, encompassing all 16 clusters of the BCT Taxonomy v1 (BCTTv1). Detailed information on BCT extraction is presented in Table 1. Among these utilized BCTs, the most frequently reported (appearing in more than 14 of the included reviews) were ‘1.1 goal setting (behaviour)’, ‘1.2 problem solving’, ‘1.4 action planning’, ‘1.5 review behaviour goals’, ‘2.2 feedback on behaviour’, ‘2.3 self-monitoring of behaviour’, ‘3.1 social support (unspecified)’, ‘4.1 instruction on how to perform the behaviour’, ‘5.1 information about health consequences’, ‘6.1 demonstration of the behaviour’, and ‘7.1 prompts/cues. Additionally, BCTs such as ‘1.3 goal setting (outcome)’, ‘8.1 behavioural practice/rehearsal’, ‘8.7 graded tasks’, ‘9.1 credible source’, ‘10.3 non-specific reward’, and ‘12.5 adding objects to the environment’ were also incorporated in more than half of the summarized reviews.

However, despite the large number of BCTs identified, only three BCTs were frequently employed in more than half of the original intervention trials included within each review: ‘1.1 goal setting (behaviour)’, ‘2.3 self-monitoring of behaviour’, and ‘4.1 instruction on how to perform the behaviour’. Moreover, a small subset of BCTs showed consistent associations with increased PA in five or more of the included reviews, specifically ‘1.1 goal setting (behaviour)’, ‘3.1 social support (unspecified)’, ‘4.1 instruction on how to perform the behaviour’, and ‘8.7 graded. In contrast, 47 of the 72 BCTs identified had no associations with increased PA. Only a few studies reported associations between certain BCTs (‘2.3 self-monitoring of behaviour’, ‘5.1 information about health consequences’, ‘5.6 information about emotional consequences’, ‘6.2 social comparison’, and ‘13.2 framing/reframing’) and reductions in PA.

### 3.5. Characteristics of BCT Application Across Different CC

#### 3.5.1. Metabolic Conditions

Two high-quality reviews with meta-analyses assessed the effectiveness of digital interventions for promoting PA in adults with metabolic conditions (Carraca et al., 2021; X. Zhang et al., 2024). Both reviews found that digital interventions led to significant PA improvement with moderate effect sizes compared to control groups, with one study (Carraca et al., 2021) showing a larger effect for in-person interventions. The most frequently applied BCTs in this population were ‘1.1 Goal setting (behaviour)’, ‘1.2 Problem solving’, ‘2.2 Feedback on behaviour’, ‘2.3 Self-monitoring of behaviour’, ‘5.1 Information about health consequences’, and ‘7.1 Prompts/cues’. Meta-regression identified five BCTs that moderated the effects of digital interventions: ‘1.1 Goal setting (behaviour)’, ‘1.3 Goal setting (outcome)’, ‘8.7 Graded tasks’, and ‘10.5 Social incentive’ were associated with positive effects, while ‘2.3 Self-monitoring of behaviour’ was linked to negative effects. Additionally, ‘8.1 Behavioural practice/rehearsal’ had significant positive moderator effects on PA in face-to-face interventions. However, the relationship between the number of BCTs and PA intervention effectiveness remains inconsistent.

#### 3.5.2. Cardiovascular Conditions

Two moderate-quality reviews conducted qualitative descriptive analyses of the effects of BCTs on PA interventions for individuals with cardiovascular conditions (Ashley et al., 2024; Duff et al., 2017). Only about half of the included studies reported significant PA improvements in both reviews. The most frequently used BCTs in the included studies were ‘1.1 Goal setting (behaviour)’, ‘2.3 Self-monitoring of behaviour’, ‘3.2 Social support (practical)’, and ‘5.1 Information about health consequences’. ‘1.1 Goal setting (behaviour)’, ‘2.2 Feedback on behaviour’, ‘4.1 Instruction on how to perform the behaviour’, and ‘5.1 Information about health consequences’ were associated with positive effects.

#### 3.5.3. Musculoskeletal Conditions

Three systematic reviews focused on promoting PA improvement and adherence in individuals with musculoskeletal conditions (Marley et al., 2017; Meade et al., 2019; Willett et al., 2019). Two of these reviews were of moderate quality, while the meta-analysis in one review was of low quality. The meta-analysis revealed small effect sizes for PA improvements in interventions lasting more than 3 months. Both moderate-quality reviews found significant PA improvements in the majority of RCTs. The most frequently used BCTs across the three studies were ‘1.1 Goal setting (behaviour)’, ‘2.3 Self-monitoring of behaviour’, ‘4.1 Instruction on how to perform the behaviour’, and ‘8.1 Behavioural practice/rehearsal’. None of the reviews employed meta-regression or subgroup analyses to quantify the relationship between the BCT categories and PA improvements, but probabilistic descriptions suggested that ‘1.1 Goal setting (behaviour)’, ‘2.3 Self-monitoring of behaviour’, ‘3.1 Social support (unspecified)’, and ‘4.1 Instruction on how to perform the behaviour’ were more consistently associated with PA improvements.

#### 3.5.4. Mixed/General Chronic Conditions

One high-quality meta-analysis examined the effects of wearable devices on PA interventions, finding small to moderate significant effect sizes (de Leeuwerk et al., 2022). Another moderate-quality systematic review highlighted the effectiveness of planned interventions combined with self-efficacy and self-regulation strategies for promoting PA in individuals with conditions such as obesity, type 2 diabetes, and cardiovascular diseases (Lin et al., 2022). Both reviews conducted comprehensive analyses of the BCTs used in the interventions, revealing high overlap in the BCTs applied. The most frequently used BCTs in both reviews were ‘1.1 Goal setting (behaviour)’, ‘2.3 Self-monitoring of behaviour’, and ‘12.5 Adding objects to the environment’. Subgroup analysis in the meta-analysis indicated that interventions using more than seven BCTs yielded higher effect sizes. ‘1.1 Goal setting (behaviour)’ was consistently associated with PA improvements across two reviews.

#### 3.5.5. Cancer

Nine systematic reviews synthesized the effects of PA interventions for cancer survivors or patients (Agirre-Elordui et al., 2024; Cooper et al., 2023; Ester et al., 2021; Finne et al., 2018; Grimmett et al., 2019; Hailey et al., 2022; Hallward et al., 2020; Mbous et al., 2020; Meyer-Schwickerath et al., 2022), with five conducting meta-analyses (Agirre-Elordui et al., 2024; Finne et al., 2018; Grimmett et al., 2019; Mbous et al., 2020; Meyer-Schwickerath et al., 2022). Four were of moderate quality, and four were of low quality, with only one being of high quality. All meta-analyses consistently reported significant minor effects of interventions on PA, with effect sizes ranging from 0.22 to 0.28. Four reviews also described PA intervention effects, with both Ester et al. (2021) and Cooper et al. (2023) finding that slightly more than half of the included studies observed positive effects of the PA interventions. However, other reviews found substantial variability in PA intervention outcomes. For instance, Hallward et al. found PA improvements in only 6 out of 15 studies (Hallward et al., 2020), while Hailey et al. observed significant PA increases in 24 out of 27 studies (Hailey et al., 2022). Frequent BCTs applied in these studies included ‘1.1 Goal setting (behaviour)’, ‘1.2 Problem solving’, ‘2.3 Self-monitoring of behaviour’, ‘3.1 Social support (unspecified)’, ‘4.1 Instruction on how to perform the behaviour’, and ‘12.5 Adding objects to the environment’. While BCTs associated with PA increases were identified, results were inconsistent and scattered. Only ‘1.1 Goal setting (behaviour)’, ‘1.4 Action planning’, and ‘8.7 Graded tasks’ were consistently associated with PA improvement in more than two included reviews. Moreover, the relationship between the number of BCTs used and the effectiveness of PA interventions varied across the included reviews.

## 4. Discussion

### 4.1. Overview of Key Findings on BCT Effectiveness

This umbrella review synthesized evidence from 18 systematic reviews on PA interventions across chronic conditions and identified two key findings. First, a set of 11 BCTs emerged as the most frequently used across interventions: ‘1.1 goal setting (behaviour)’, ‘1.2 problem solving’, ‘2.2 feedback on behaviour’, ‘2.3 self-monitoring of behaviour’, ‘4.1 instruction on how to perform the behaviour’, ‘1.4 action planning’, ‘1.5 review behaviour goals’, ‘5.1 information about health consequences’, ‘6.1 demonstration of the behaviour’, ‘3.1 social support (unspecified)’, and ‘7.1 prompts/cues’. These reflect commonly adopted strategies in PA promotion across diverse chronic condition populations. Second, among these, only four BCTs were consistently associated with increased PA in five or more of the included reviews: ‘1.1 goal setting (behaviour)’, ‘3.1 social support (unspecified)’, ‘4.1 instruction on how to perform the behaviour’, and ‘8.7 graded tasks’. These findings suggest that while many BCTs are utilized, only a limited subset is consistently associated with improved PA outcomes across conditions, highlighting the importance of strategic selection and application of BCTs in intervention design.

Notably, most interventions combined multiple BCTs, with effective programs frequently incorporating multicomponent, self-regulatory strategies. The consistent use of such multifaceted approaches reinforces existing evidence that no single technique is sufficient in isolation. Interventions tend to be more successful or show stronger associations when they integrate complementary strategies targeting motivation, self-regulation, and social support (Knittle et al., 2018; Pynnonen et al., 2023; Tang et al., 2025). For example, Michie et al. found in a meta-regression that interventions were significantly more effective when self-monitoring was combined with at least one other self-regulatory BCT, such as goal setting or feedback (Michie et al., 2009). This pattern is consistent with our findings that the BCTs most consistently linked to positive outcomes—goal setting, graded tasks, instruction, and social support—are typically combined to facilitate behaviour change. These findings are also consistent with Control Theory, which emphasizes a cyclical process of goal setting, behavioural monitoring, feedback, and adjustment as central to effective behaviour regulation (Carver & Scheier, 1998; Gude & Peek, 2019). Our results underscore that successful PA interventions for chronic conditions tend to employ integrated BCT packages that support individuals in setting achievable goals, building confidence and skills, receiving social reinforcement, and progressing gradually. However, the observed heterogeneity in outcomes suggests that more BCTs do not necessarily translate into greater effectiveness. While Lin et al. (2022) reported that interventions with more than seven BCTs achieved larger effect sizes, others found no consistent dose–response pattern. Thus, while multicomponent strategies are generally beneficial, the relevance and quality of selected BCTs may be more critical than their sheer number (Hagger et al., 2020).

### 4.2. Condition-Specific BCT Application

Findings from this review indicate that specific chronic condition groups tend to benefit from distinct, commonly used BCTs. Tailoring PA interventions by incorporating the most appropriate BCTs for each condition type enhances intervention effectiveness (Ma et al., 2022). BCTs’ differential use and relevance across conditions likely reflect variations in disease characteristics, symptom burden, and behavioural barriers.

For individuals with metabolic conditions (e.g., obesity or type 2 diabetes), ‘8.7 graded tasks’ and ‘10.5 social incentive’ were frequently used and positively associated with PA improvement. These techniques are particularly relevant for individuals who often face motivational inertia or physical limitations, as they support gradual engagement and reinforce behaviour through external rewards (Michaelsen & Esch, 2021). However, ‘2.3 self-monitoring of behaviour’ was negatively associated with PA in some digital interventions, suggesting that tracking alone, without feedback or encouragement, may undermine motivation (Carraca et al., 2021).

In cardiovascular conditions, BCTs such as ‘4.1 instruction on how to perform the behaviour’ and high-frequency use of ‘5.1 information about health consequences’ were prominent. These BCTs help address safety concerns and exercise-related anxiety common in this population by enhancing self-efficacy through knowledge and guided practice (Duff et al., 2017).

Musculoskeletal interventions consistently employed ‘3.1 social support (unspecified)’ and ‘4.1 instruction on how to perform the behaviour’, positively associated with increased PA. These techniques address key psychological and functional barriers in this population, such as kinesiophobia, low self-efficacy, and restricted mobility, by enhancing individuals’ confidence, safety perception, and sense of support (Webb et al., 2022). Instruction provides step-by-step guidance on movement execution, reducing uncertainty and physical hesitation. Meanwhile, social support mechanisms foster accountability, emotional encouragement, and shared engagement, creating a motivational climate conducive to sustained participation (J. Zhang et al., 2016). This combined approach is especially relevant for individuals managing chronic musculoskeletal conditions, where pain, disuse, and fear often inhibit active lifestyles (Stevens et al., 2020). The observed effectiveness of these techniques aligns with Self-Determination Theory, which posits that behaviour change is more likely when individuals experience both competence and relatedness, outcomes that are directly supported by instruction and social reinforcement (Gillison et al., 2019).

In interventions addressing mixed or multiple chronic conditions, ‘1.1 goal setting (behaviour)’ was the only BCT consistently associated with increased PA. These findings suggest that goal setting offers a flexible and scalable strategy to support behaviour change in populations characterized by diverse and overlapping health challenges. Its capacity to accommodate varying functional baselines and personalized objectives makes it particularly suitable for heterogeneous groups, where standardized approaches may fall short (Mozafarinia et al., 2024).

Among cancer survivors, ‘1.4 Action planning’ and ‘8.7 Graded tasks’ emerged as the most consistent BCTs associated with PA improvements. These techniques are particularly well-suited to the complex physical and psychological challenges commonly experienced in the post-treatment phase, including persistent fatigue, reduced functional capacity, and uncertainty regarding safe levels of exertion (Grimmett et al., 2019; Meyer-Schwickerath et al., 2022). Action planning facilitates the translation of intentions into structured, context-specific behavioural strategies by prompting individuals to specify when, where, and how PA will be undertaken (Sniehotta et al., 2005). This level of detail helps survivors overcome decision paralysis and anticipatory anxiety by integrating PA into their daily routines realistically and sustainably. Graded tasks, in turn, enable a progressive approach to physical reconditioning by breaking down activity goals into manageable and incrementally challenging steps (Meyer-Schwickerath et al., 2022). This technique supports mastery experiences, fosters self-efficacy, and accommodates fluctuating energy levels, which are common among cancer survivors (Voss et al., 2024). When combined, action planning and graded tasks provide the strategic framework and the adaptive progression necessary to support engagement, reduce psychological resistance, and promote sustained PA behaviour in this population.

### 4.3. Integration and Implications

This umbrella review underscores that effective PA interventions for chronic conditions consistently incorporate self-regulatory and multicomponent BCTs tailored to the needs of specific populations. Techniques such as ‘1.1 goal setting (behaviour)’, ‘4.1 instruction on how to perform the behaviour’, ‘3.1 social support (unspecified)’, and ‘8.7 graded tasks’ align closely with Control Theory (Carver & Scheier, 1998), which emphasizes goal-directed behaviour, feedback loops, and adjustment. Interventions combining these elements foster self-monitoring, adaptive planning, and sustained motivation—key for long-term adherence.

Similarly, our condition-specific findings reflect Social Cognitive Theory (Bandura, 2013), particularly constructs like self-efficacy and outcome expectations. For instance, cardiac patients benefit from instructional support that alleviates fear; musculoskeletal patients require mastery experiences to overcome movement-related anxiety; metabolic patients respond well to socially reinforced, graded approaches. Previous meta-analyses have consistently shown that interventions integrating self-regulatory BCTs, such as goal setting, feedback, and graded tasks, are more effective in promoting PA among clinical populations (Greaves et al., 2011; Michie et al., 2009; Olander et al., 2013; Samdal et al., 2017). Moreover, combining multiple self-regulatory BCTs has yielded more potent effects on PA outcomes (Sequeira et al., 2023).

Beyond individual and theoretical mechanisms, contextual factors substantially influence both the selection and the effectiveness of BCTs. Evidence from digital and remotely delivered interventions indicates that self-regulatory techniques such as self-monitoring and feedback are more feasible and effective in technology-supported environments (Patel et al., 2022). In contrast, face-to-face or rehabilitation-based programs tend to achieve better outcomes when they emphasize social support, behavioural demonstration, and guided practice (Tamuleviciute-Prasciene et al., 2022). Moreover, intervention setting and resource context influence implementation fidelity. Programs delivered in clinical or hospital environments typically prioritize safety-related and informational BCTs, whereas community-based interventions rely more on motivational and participatory strategies that foster social connectedness (Ahmed et al., 2024; Presseau et al., 2019). These contextual influences highlight that BCT effectiveness must be interpreted within the broader delivery mode, cultural norms, and environmental conditions in which behaviour change takes place.

A significant implication is that intervention success depends not only on using multiple BCTs but also on selecting combinations that match the behavioural and contextual needs of the target population. Programs for arthritis might emphasize demonstration and habit rehearsal, while those for diabetes might prioritize self-monitoring and problem solving (Rezende et al., 2021). Moreover, sustainable PA change hinges on transitioning from structured intervention to self-managed maintenance—requiring planning, feedback, and habit-building support (Blicher-Hansen et al., 2024).

### 4.4. Strengths and Limitations of the Evidence Base

This umbrella review offers a comprehensive synthesis of BCT applications in PA interventions for chronic conditions, drawing on 18 systematic reviews and 468 primary studies. A key strength lies in its broad disease coverage—metabolic, cardiovascular, musculoskeletal, cancer, and mixed conditions—enabling cross-condition comparisons and identification of shared and specific BCT patterns. Methodologically, most of the included reviews were rated as moderate or high quality via AMSTAR 2, and standardized BCTTv1 was used to harmonize intervention content across studies, overcoming previous inconsistencies in terminology. Additionally, overlap analysis showed minimal duplication of primary trials across reviews (e.g., CCA = 0.087 for cancer), indicating that findings reflect independent bodies of evidence rather than repeated inclusion of the same influential studies.

Nonetheless, several limitations warrant caution in interpretation. First, inconsistent BCT reporting and coding across reviews posed challenges; many relied on narrative synthesis or lacked comprehensive extraction, potentially underestimating the presence or relevance of specific techniques. Intervention fidelity and dosage were also rarely reported, making it difficult to distinguish between superficially and rigorously implemented BCTs—two programs coded as using ‘goal setting,’ for example, may differ substantially in delivery and impact. Moreover, fewer than half of the reviews conducted formal moderator analyses, and comparative data between effective and ineffective interventions were often unavailable or inconsistently reported. Consequently, associations between frequently used BCTs and improvements in PA should be interpreted as descriptive and correlational rather than causal.

Beyond BCT content, intervention effectiveness is also shaped by implementation and user-centred factors. Acceptability, usability, engagement, provider competence, delivery mode, and contextual fit, particularly for digital interventions, were infrequently reported. The absence of these data limits interpretation because a lack of effect may reflect suboptimal delivery rather than the BCTs employed.

The evidence base also exhibits notable gaps. Underrepresented populations included older adults, socioeconomically disadvantaged groups, and individuals from low- and middle-income countries. Similarly, chronic respiratory diseases and mental health comorbidities were largely absent from the included reviews. These gaps constrain the generalizability of findings to diverse real-world populations. Furthermore, the review excluded grey literature, which may have introduced publication and language bias. Although approximately half of the included reviews formally assessed publication bias and found minimal evidence of it, these assessments were not consistent across all studies, and residual bias cannot be excluded. In addition, heterogeneity in intervention types, outcomes, and follow-up periods further complicates synthesis, and the presence of five low-quality reviews raises questions about the reliability of some conclusions.

Finally, this review interpreted the observed BCT patterns in light of established behaviour change theories to enhance conceptual understanding. However, these theoretical links remain interpretive rather than empirically tested, as the included reviews primarily described the presence and frequency of BCTs without directly examining underlying mechanisms. Therefore, the associations between specific BCTs and theoretical constructs should be viewed as explanatory rather than causal, and future research should more explicitly integrate and evaluate theory-based pathways.

## 5. Conclusions

This review offers the first comprehensive synthesis of evidence on the application of BCTs and their associations with PA outcomes in interventions for individuals with chronic conditions. This review provides a valuable foundation for developing more targeted, theory-driven, and scalable interventions by identifying consistently effective strategies across multiple disease contexts. The findings highlight the importance of incorporating multicomponent and self-regulatory approaches tailored to the unique challenges faced by different chronic condition populations. These insights have important implications for clinical practice, public health program design, and behaviour change policymaking. Moreover, as healthcare systems increasingly adopt digital and remote delivery models, integrating behaviourally informed techniques becomes even more essential. Future research should prioritize rigorously designed trials that explore the contextual effectiveness of different strategies, including underrepresented populations and delivery modes. Establishing a clearer evidence base for what works, for whom, and under which conditions will be key to improving PA engagement and long-term health outcomes among people with chronic diseases.

## Figures and Tables

**Table 1:** BCT Application Matrix.

BCT	Metabolic Conditions		Cardiovascular Conditions		Musculoskeletal Conditions			Mixed/General Chronic Conditions		Cancer									✓	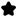	▲	▼
	(X. Zhang et al., 2024)	(Carraca et al., 2021)	(Ashley et al., 2024)	(Duff et al., 2017)	(Marley et al., 2017)	(Meade et al., 2019)	(Willett et al., 2019)	(de Leeuwerk et al., 2022)	(Lin et al., 2022)	(Agirre-Elordui et al., 2024)	(Cooper et al., 2023)	(Ester et al., 2021)	(Finne et al., 2018)	(Grimmett et al., 2019)	(Hailey et al., 2022)	(Hallward et al., 2020)	(Mbous et al., 2020)	(Meyer-Schwickerath et al., 2022)				
1.1	✓ 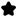	✓ 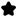 ▲		✓ 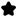 ▲	✓ 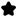 ▲	✓▲	✓ 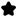 ▲	✓ 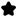 ▲	✓ 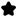 ▲	✓ 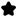	✓ 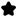 ▲	✓ 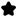 ▲	✓ 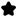	✓ 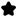	✓ 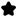 ▲	✓	✓ 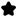	✓ 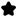	17	15	10	0
1.2	✓ 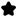	✓ 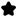	✓	✓		✓	✓	✓	✓ 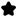	✓ 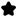	✓	✓ 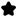	✓ 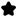	✓ 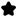	✓	✓	✓ 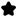	✓ 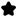	17	9	0	0
1.3	✓	✓▲		✓		✓	✓	✓		✓ 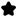			✓	✓			✓ 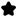	✓	11	2	1	0
1.4	✓ 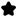	✓		✓		✓	✓ 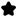	✓ 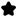	✓	✓ 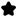	✓	✓▲	✓	✓ 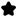 ▲	✓	✓	✓ 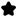	✓▲	16	6	3	0
1.5	✓ 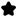	✓	✓	✓		✓	✓	✓	✓	✓ 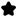		✓ 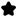	✓	✓	✓	✓	✓	✓	16	3	0	0
1.6	✓	✓				✓		✓	✓		✓		✓	✓			✓		9	0	0	0
1.7	✓			✓		✓			✓		✓						✓	✓	7	0	0	0
1.8		✓		✓		✓	✓▲		✓				✓						6	0	1	0
1.9		✓				✓			✓				✓	✓					5	0	0	0
2.1		✓		✓		✓	✓				✓		✓		✓				7	0	0	0
2.2	✓ 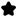	✓ 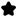	✓	✓▲		✓	✓	✓ 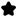	✓	✓ 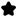	✓ 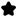	✓ 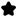	✓	✓	✓	✓	✓ 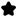	✓ 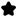	17	8	1	0
2.3	✓ 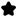	✓ 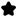 ▼		✓ 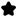	✓ 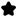 ▲	✓	✓ 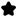 ▲	✓ 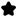	✓ 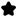	✓ 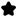	✓ 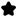 ▲	✓ 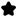	✓ 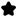	✓ 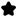	✓▲	✓ 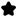	✓ 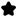	✓ 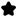	17	15	4	1
2.4	✓	✓ 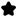		✓				✓	✓						✓		✓		7	1	0	0
2.5		✓		✓											✓				3	0	0	0
2.6				✓		✓			✓					✓	✓	✓			6	0	0	0
2.7		✓		✓			✓	✓	✓					✓				✓	7	0	0	0
3.1	✓	✓ 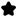		✓		✓▲	✓▲	✓	✓ 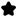 ▲		✓ 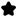	✓ 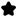	✓ 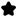	✓ 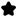 ▲	✓	✓ 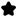 ▲	✓	✓ 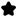	15	8	5	0
3.2	✓			✓ 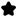					✓▲	✓			✓	✓	✓	✓	✓		9	1	1	0
3.3	✓	✓		✓						✓ 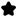	✓				✓		✓		7	1	0	0
4.1	✓	✓ 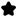	✓▲	✓▲	✓ 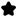 ▲	✓ 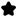 ▲	✓ 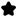	✓▲	✓ 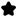	✓ 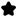	✓	✓ 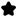	✓ 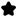	✓ 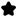	✓ 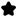	✓ 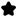 ▲		✓ 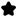	17	12	6	0
4.2		✓					✓							✓		✓▲	✓		5	0	1	0
4.3	✓																		1	0	0	0
5.1	✓ 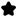	✓ 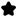	✓	✓ 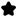 ▲		✓	✓	✓▲	✓ 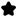	✓ 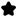	✓	✓ 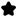	✓▼	✓ 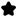	✓		✓	✓	16	7	2	1
5.2	✓												✓	✓					3	0	0	0
5.3							✓	✓▲	✓	✓			✓	✓		✓▲	✓		8	2	0	0
5.4		✓				✓													2	0	0	0
5.6		✓							✓				✓▼	✓					4	0	0	1
6.1	✓	✓	✓	✓		✓ 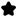 ▲	✓		✓	✓ 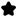 ▲	✓		✓	✓	✓	✓	✓	✓ 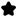	15	3	2	0
6.2		✓		✓		✓▲		✓▲	✓				✓▼		✓				7	0	2	1
6.3		✓																	1	0	0	0
7.1	✓ 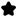	✓ 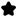		✓		✓		✓	✓	✓ 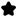	✓	✓ 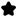	✓▲	✓	✓	✓▲	✓	✓	15	4	2	0
7.3													✓▲						1	0	1	0
8.1		✓ 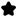 ▲	✓		✓ 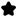	✓ 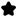 ▲	✓ 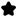			✓ 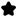	✓	✓ 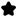	✓ 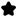	✓	✓	✓ 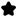	✓	✓	14	8	2	0
8.2									✓				✓				✓		3	0	0	0
8.3	✓								✓		✓	✓ 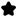							4	1	0	0
8.4	✓																	✓▲	2	0	1	0
8.6						✓	✓ 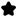							✓		✓ 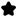	✓		5	2	0	0
8.7	✓	✓ 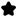 ▲		✓		✓	✓ 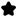	✓ 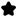		✓ 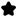	✓		✓▲	✓ 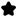 ▲		✓	✓▲	✓ 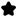 ▲	13	6	5	0
9.1		✓ 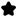		✓		✓	✓ 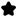		✓	✓ 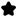	✓	✓ 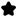	✓	✓ 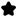	✓	✓▲		✓▲	13	5	2	0
9.2	✓	✓							✓	✓ 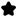	✓		✓	✓				✓	8	1	0	0
9.3		✓				✓													2	0	0	0
10.1		✓													✓				2	0	0	0
10.2		✓														✓			2	0	0	0
10.3	✓	✓		✓		✓	✓	✓	✓		✓		✓▲			✓			10	0	1	0
10.4		✓		✓		✓		✓	✓		✓		✓▲	✓	✓				9	0	1	0
10.5		✓▲							✓										2	0	1	0
10.6		✓									✓								2	0	0	0
10.7		✓																	1	0	0	0
10.8		✓																	1	0	0	0
10.9		✓				✓▲		✓▲			✓		✓					✓	6	0	2	0
10.11						✓													1	0	0	0
11.1				✓					✓										2	0	0	0
11.2		✓		✓			✓						✓	✓				✓	6	0	0	0
12.1				✓					✓	✓ 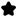 ▲	✓		✓		✓				6	1	1	0
12.2		✓							✓										2	0	0	0
12.3		✓											✓						2	0	0	0
12.4		✓					✓												2	0	0	0
12.5		✓		✓		✓	✓	✓ 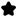	✓ 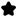	✓ 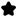		✓ 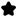	✓ 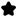	✓ 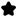	✓ 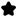 ▲	✓ 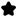 ▲		✓ 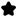	13	9	2	0
12.6						✓	✓ 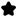												2	1	0	0
13.1		✓																	1	0	0	0
13.2	✓	✓				✓			✓	✓ 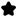 ▼			✓	✓				✓	8	1	0	1
13.4	✓	✓																	2	0	0	0
13.5		✓									✓		✓						3	0	0	0
14.1		✓																	1	0	0	0
14.4		✓																	1	0	0	0
14.6																	✓		1	0	0	0
14.8																	✓		1	0	0	0
15.1	✓	✓							✓	✓ 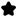	✓			✓					6	1	0	0
15.3		✓																	1	0	0	0
15.4	✓	✓					✓		✓		✓		✓						6	0	0	0
16.2													✓						1	0	0	0
16.3		✓																	1	0	0	0

Notes: BCT = behaviour change technique (coded according to the Behaviour Change Technique Taxonomy v1): 1.1 Goal setting (behaviour); 1.2 Problem solving; 1.3 Goal setting (outcome); 1.4 Action planning; 1.5 Review behaviour goal(s); 1.6 Discrepancy between current behaviour and goal; 1.7 Review outcome goal(s); 1.8 Behavioural contract; 1.9 Commitment; 2.1 Monitoring of behaviour by others without feedback; 2.2 Feedback on behaviour; 2.3 Self-monitoring of behaviour; 2.4 Self-monitoring of outcome(s) of behaviour; 2.5 Monitoring of outcome(s) of behaviour without feedback; 2.6 Biofeedback; 2.7 Feedback on outcome(s) of behaviour; 3.1 Social support (unspecified); 3.2 Social support (practical); 3.3 Social support (emotional); 4.1 Instruction on how to perform the behaviour; 4.2 Information about antecedents; 4.3 Re-attribution; 5.1 Information about health consequences; 5.2 Salience of consequences; 5.3 Information about social and environmental consequences; 5.4 Monitoring of emotional consequences; 5.6 Information about emotional consequences; 6.1 Demonstration of the behaviour; 6.2 Social comparison; 6.3 Information about others’ approval; 7.1 Prompts/cues; 7.3 Reduce prompts/cues; 8.1 Behavioural practice/rehearsal; 8.2 Behaviour substitution; 8.3 Habit formation; 8.4 Habit reversal; 8.6 Generalization of target behaviour; 8.7 Graded tasks; 9.1 Credible source; 9.2 Pros and cons; 9.3 Comparative imagining of future outcomes; 10.1 Material incentive (behaviour); 10.2 Material reward (behaviour); 10.3 Non-specific reward; 10.4 Social reward; 10.5 Social incentive; 10.6 Non-specific incentive; 10.7 Self-incentive; 10.8 Incentive (outcome); 10.9 Self-reward; 10.11 Reward (outcome); 11.1 Pharmacological support; 11.2 Reduce negative emotions; 12.1 Restructuring the physical environment; 12.2 Restructuring the social environment; 12.3 Avoidance/reducing exposure to cues for the behaviour; 12.4 Distraction; 12.5 Adding objects to the environment; 12.6 Body changes; 13.1 Identification of self as role model; 13.2 Framing/reframing; 13.4 Valued self-identity; 13.5 Identity associated with changed behaviour; 14.1 Behaviour cost; 14.4 Reward approximation; 14.6 Situation-specific reward; 14.8 Reward alternative behaviour; 15.1 Verbal persuasion about capability; 15.3 Focus on past success; 15.4 Self-talk; 16.2 Imaginary reward; 16.3 Vicarious consequences. ✓ = BCTs that were identified in included studies; 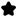 = BCTs that were frequently used (reported in ≥50% of included studies); ▲ = BCTs positively associated with improvements in physical activity; ▼ = BCTs negatively associated with improvements in physical activity.

## Data Availability

All data supporting the findings of this umbrella review are available within the article and its Supplementary Materials. Extracted data tables, the data collection form, and quality assessment summaries can be obtained from the corresponding author upon reasonable request. No new primary data or analytic code was generated.

## Supplementary Materials

The following supporting information can be downloaded at https://www.mdpi.com/article/10.3390/bs15111448/s1, S1: Searching Strategies; S2: Quality Appraisal; S3: Characteristics of Included Studies; S4: Overlap Matrix.

## References

[B1-behavsci-15-01448] Agirre-Elordui S., Fernandez-Landa J., Olasagasti-Ibargoien J., Castaneda-Babarro A. (2024). Physical activity maintenance in colorectal cancer survivors after an exercise intervention applying behaviour change techniques: A systematic review and meta-analysis. Journal of Cancer Survivorship.

[B2-behavsci-15-01448] Ahmed S., Lazo Green K., McGarrigle L., Money A., Pendleton N., Todd C. (2024). Interventions based on behaviour change techniques to encourage physical activity or decrease sedentary behaviour in community-dwelling adults aged 50–70: Systematic review with intervention component analysis. Journal of Aging and Physical Activity.

[B3-behavsci-15-01448] Alqahtani S. (2022). Are Us Adults with Chronic Health Conditions Meeting Public Health Recommendations For Physical Activity?. Medicine & Science in Sports & Exercise.

[B4-behavsci-15-01448] Anderson E., Durstine J. L. (2019). Physical activity, exercise, and chronic diseases: A brief review. Sports Medicine and Health Science.

[B5-behavsci-15-01448] Aromataris E., Fernandez R., Godfrey C., Holly C., Khalil H., Tungpunkom P., Aromataris E., Munn Z. (2020). Chapter 10: Umbrella reviews. Joanna Briggs Institute reviewer’s manual.

[B6-behavsci-15-01448] Ashley K., Tang M. Y., Flynn D., Cooper M., Errington L., Avery L. (2024). Identifying the active ingredients of training interventions for healthcare professionals to promote and support increased levels of physical activity in adults with heart failure: A systematic review. Health Psychology Review.

[B7-behavsci-15-01448] Bandura A. (2013). Health promotion from the perspective of social cognitive theory. Understanding and changing health behaviour.

[B8-behavsci-15-01448] Beaglehole R., Bonita R., Horton R., Adams C., Alleyne G., Asaria P., Baugh V., Bekedam H., Billo N., Casswell S., Cecchini M., Colagiuri R., Colagiuri S., Collins T., Ebrahim S., Engelgau M., Galea G., Gaziano T., Geneau R., Alliance N. C. D. (2011). Priority actions for the non-communicable disease crisis. The Lancet.

[B9-behavsci-15-01448] Blicher-Hansen J., Chilcot J., Gardner B. (2024). Experiences of successful physical activity maintenance among adults with type 2 diabetes: A theory-based qualitative study. Psychology & Health.

[B10-behavsci-15-01448] Bull F. C., Al-Ansari S. S., Biddle S., Borodulin K., Buman M. P., Cardon G., Carty C., Chaput J. P., Chastin S., Chou R., Dempsey P. C., DiPietro L., Ekelund U., Firth J., Friedenreich C. M., Garcia L., Gichu M., Jago R., Katzmarzyk P. T., Willumsen J. F. (2020). World Health Organization 2020 guidelines on physical activity and sedentary behaviour. British Journal of Sports Medicine.

[B11-behavsci-15-01448] Carraca E., Encantado J., Battista F., Beaulieu K., Blundell J., Busetto L., van Baak M., Dicker D., Ermolao A., Farpour-Lambert N., Pramono A., Woodward E., Bellicha A., Oppert J. M. (2021). Effective behaviour change techniques to promote physical activity in adults with overweight or obesity: A systematic review and meta-analysis. Obesity Reviews.

[B12-behavsci-15-01448] Carver C., Scheier M. (1998). On the self-regulation of behaviour.

[B13-behavsci-15-01448] Cooper K. B., Lapierre S., Carrera Seoane M., Lindstrom K., Pritschmann R., Donahue M., Christou D. D., McVay M. A., Jake-Schoffman D. E. (2023). Behaviour change techniques in digital physical activity interventions for breast cancer survivors: A systematic review. Translational Behavioral Medicine.

[B14-behavsci-15-01448] de Leeuwerk M. E., Bor P., van der Ploeg H. P., de Groot V., van der Schaaf M., van der Leeden M., consortium O. (2022). The effectiveness of physical activity interventions using activity trackers during or after inpatient care: A systematic review and meta-analysis of randomized controlled trials. International Journal of Behavioral Nutrition and Physical Activity.

[B15-behavsci-15-01448] Duff O. M., Walsh D. M., Furlong B. A., O’Connor N. E., Moran K. A., Woods C. B. (2017). Behaviour change techniques in physical activity eHealth interventions for people with cardiovascular disease: Systematic review. Journal of Medical Internet Research.

[B16-behavsci-15-01448] Ester M., Eisele M., Wurz A., McDonough M. H., McNeely M., Culos-Reed S. N. (2021). Current evidence and directions for future research in eHealth physical activity interventions for adults affected by cancer: Systematic review. JMIR Cancer.

[B17-behavsci-15-01448] Finne E., Glausch M., Exner A. K., Sauzet O., Stolzel F., Seidel N. (2018). Behaviour change techniques for increasing physical activity in cancer survivors: A systematic review and meta-analysis of randomized controlled trials. Cancer Management and Research.

[B18-behavsci-15-01448] Forechi L., Mill J. G., Griep R. H., Santos I., Pitanga F., Molina M. (2018). Adherence to physical activity in adults with chronic diseases: ELSA-Brasil. Revista de Saúde Pública.

[B19-behavsci-15-01448] GBD 2019 Diseases and Injuries Collaborators (2020). Global burden of 369 diseases and injuries in 204 countries and territories, 1990–2019: A systematic analysis for the Global Burden of Disease Study 2019. Lancet.

[B20-behavsci-15-01448] GBD 2019 Risk Factors Collaborators (2020). Global burden of 87 risk factors in 204 countries and territories, 1990–2019: A systematic analysis for the Global Burden of Disease Study 2019. The Lancet.

[B21-behavsci-15-01448] Gillison F. B., Rouse P., Standage M., Sebire S. J., Ryan R. M. (2019). A meta-analysis of techniques to promote motivation for health behaviour change from a self-determination theory perspective. Health Psychology Review.

[B22-behavsci-15-01448] Greaves C. J., Sheppard K. E., Abraham C., Hardeman W., Roden M., Evans P. H., Schwarz P., Group I. S. (2011). Systematic review of reviews of intervention components associated with increased effectiveness in dietary and physical activity interventions. BMC Public Health.

[B23-behavsci-15-01448] Grimmett C., Corbett T., Brunet J., Shepherd J., Pinto B. M., May C. R., Foster C. (2019). Systematic review and meta-analysis of maintenance of physical activity behaviour change in cancer survivors. International Journal of Behavioral Nutrition and Physical Activity.

[B24-behavsci-15-01448] Gude W. T., Peek N. (2019). Control theory to design and evaluate audit and feedback interventions. Applied interdisciplinary theory in health informatics.

[B25-behavsci-15-01448] Hagger M. S., Moyers S., McAnally K., McKinley L. E. (2020). Known knowns and known unknowns on behaviour change interventions and mechanisms of action. Health Psychology Review.

[B26-behavsci-15-01448] Hailey V., Rojas-Garcia A., Kassianos A. P. (2022). A systematic review of behaviour change techniques used in interventions to increase physical activity among breast cancer survivors. Breast Cancer.

[B27-behavsci-15-01448] Hallward L., Patel N., Duncan L. R. (2020). Behaviour change techniques in physical activity interventions for men with prostate cancer: A systematic review. Journal of Health Psychology.

[B28-behavsci-15-01448] Kirvalidze M., Abbadi A., Dahlberg L., Sacco L. B., Calderon-Larranaga A., Morin L. (2023). Estimating pairwise overlap in umbrella reviews: Considerations for using the corrected covered area (CCA) index methodology. Research Synthesis Methods.

[B29-behavsci-15-01448] Knittle K., Nurmi J., Crutzen R., Hankonen N., Beattie M., Dombrowski S. U. (2018). How can interventions increase motivation for physical activity? A systematic review and meta-analysis. Health Psychology Review.

[B30-behavsci-15-01448] Lau Y., Chew H. S. J., Ang W. H. D., Ang W. W., Yeo C. Y., Lim G. Z. Q., Wong S. H., Lau S. T., Cheng L. J. (2024). Effects of digital health interventions on the psychological outcomes of perinatal women: Umbrella review of systematic reviews and meta-analyses. Health Psychology Review.

[B31-behavsci-15-01448] Lin H., Xu D., Yang M., Ma X., Yan N., Chen H., He S., Deng N. (2022). Behaviour change techniques that constitute effective planning interventions to improve physical activity and diet behaviour for people with chronic conditions: A systematic review. BMJ Open.

[B32-behavsci-15-01448] Ma J. K., Ramachandran S., Sandhu A., Tsui K., Hoens A., Hu D., Li L. C. (2022). Tailored interventions for supporting physical activity participation in people with arthritis and related conditions: A systematic review. Current Treatment Options in Rheumatology.

[B33-behavsci-15-01448] Marley J., Tully M. A., Porter-Armstrong A., Bunting B., O’Hanlon J., Atkins L., Howes S., McDonough S. M. (2017). The effectiveness of interventions aimed at increasing physical activity in adults with persistent musculoskeletal pain: A systematic review and meta-analysis. BMC Musculoskeletal Disorders.

[B34-behavsci-15-01448] Mbous Y. P., Patel J., Kelly K. M. (2020). A systematic review and meta-analysis of physical activity interventions among colorectal cancer survivors. Translational Behavioral Medicine.

[B35-behavsci-15-01448] Meade L. B., Bearne L. M., Sweeney L. H., Alageel S. H., Godfrey E. L. (2019). Behaviour change techniques associated with adherence to prescribed exercise in patients with persistent musculoskeletal pain: Systematic review. British Journal of Health Psychology.

[B36-behavsci-15-01448] Meyer-Schwickerath C., Morawietz C., Baumann F. T., Huber G., Wiskemann J. (2022). Efficacy of face-to-face behaviour change counseling interventions on physical activity behaviour in cancer survivors—A systematic review and meta-analysis. Disability and Rehabilitation.

[B37-behavsci-15-01448] Michaelsen M. M., Esch T. (2021). Motivation and reward mechanisms in health behaviour change processes. Brain Research.

[B38-behavsci-15-01448] Michie S., Abraham C., Whittington C., McAteer J., Gupta S. (2009). Effective techniques in healthy eating and physical activity interventions: A meta-regression. Health Psychology.

[B39-behavsci-15-01448] Michie S., Ashford S., Sniehotta F. F., Dombrowski S. U., Bishop A., French D. P. (2011). A refined taxonomy of behaviour change techniques to help people change their physical activity and healthy eating behaviours: The CALO-RE taxonomy. Psychology & Health.

[B40-behavsci-15-01448] Michie S., Richardson M., Johnston M., Abraham C., Francis J., Hardeman W., Eccles M. P., Cane J., Wood C. E. (2013). The behaviour change technique taxonomy (v1) of 93 hierarchically clustered techniques: Building an international consensus for the reporting of behaviour change interventions. Annals of Behavioral Medicine.

[B41-behavsci-15-01448] Mozafarinia M., Mate K. K. V., Brouillette M. J., Fellows L. K., Knauper B., Mayo N. E. (2024). An umbrella review of the literature on the effectiveness of goal setting interventions in improving health outcomes in chronic conditions. Disability and Rehabilitation.

[B42-behavsci-15-01448] Murray J. M., Brennan S. F., French D. P., Patterson C. C., Kee F., Hunter R. F. (2017). Effectiveness of physical activity interventions in achieving behaviour change maintenance in young and middle aged adults: A systematic review and meta-analysis. Social Science & Medicine.

[B43-behavsci-15-01448] Olander E. K., Fletcher H., Williams S., Atkinson L., Turner A., French D. P. (2013). What are the most effective techniques in changing obese individuals’ physical activity self-efficacy and behaviour: A systematic review and meta-analysis. International Journal of Behavioral Nutrition and Physical Activity.

[B44-behavsci-15-01448] Page M. J., McKenzie J. E., Bossuyt P. M., Boutron I., Hoffmann T. C., Mulrow C. D., Shamseer L., Tetzlaff J. M., Akl E. A., Brennan S. E., Chou R., Glanville J., Grimshaw J. M., Hrobjartsson A., Lalu M. M., Li T., Loder E. W., Mayo-Wilson E., McDonald S., Moher D. (2021). The PRISMA 2020 statement: An updated guideline for reporting systematic reviews. BMJ.

[B45-behavsci-15-01448] Patel M. L., Cleare A. E., Smith C. M., Rosas L. G., King A. C. (2022). Detailed versus simplified dietary self-monitoring in a digital weight loss intervention among racial and ethnic minority adults: Fully remote, randomized pilot study. JMIR Formative Research.

[B46-behavsci-15-01448] Pedersen B. K., Saltin B. (2015). Exercise as medicine—Evidence for prescribing exercise as therapy in 26 different chronic diseases. Scandinavian Journal of Medicine & Science in Sports.

[B47-behavsci-15-01448] Piercy K. L., Troiano R. P., Ballard R. M., Carlson S. A., Fulton J. E., Galuska D. A., George S. M., Olson R. D. (2018). The physical activity guidelines for Americans. JAMA.

[B48-behavsci-15-01448] Pollock M., Fernandes R. M., Pieper D., Tricco A. C., Gates M., Gates A., Hartling L. (2019). Preferred Reporting Items for Overviews of Reviews (PRIOR): A protocol for development of a reporting guideline for overviews of reviews of healthcare interventions. Systematic Reviews.

[B49-behavsci-15-01448] Presseau J., Ivers N. M., Newham J. J., Knittle K., Danko K. J., Grimshaw J. M. (2015). Using a behaviour change techniques taxonomy to identify active ingredients within trials of implementation interventions for diabetes care. Implementation Science.

[B50-behavsci-15-01448] Presseau J., McCleary N., Lorencatto F., Patey A. M., Grimshaw J. M., Francis J. J. (2019). Action, actor, context, target, time (AACTT): A framework for specifying behaviour. Implementation Science.

[B51-behavsci-15-01448] Pynnonen K., Hassandra M., Tolvanen A., Siltanen S., Portegijs E., Rantanen T. (2023). Do the integrated theories of self-determination and planned behaviour explain the change in active life engagement following a motivational counseling intervention among older people?. Social Science & Medicine.

[B52-behavsci-15-01448] Rezende M., Brito N., Farias F., Silva C., Cernigoy C., da Silva J. R., Moreira M., Santana O., Hissadomi M., Frucchi R. (2021). Improved function and strength in patients with knee osteoarthritis as a result of adding a two-day educational program to usual care. Prospective randomized trial. Osteoarthritis and Cartilage Open.

[B53-behavsci-15-01448] Rhodes R. E., Dickau L. (2012). Experimental evidence for the intention-behaviour relationship in the physical activity domain: A meta-analysis. Health Psychology.

[B54-behavsci-15-01448] Samdal G. B., Eide G. E., Barth T., Williams G., Meland E. (2017). Effective behaviour change techniques for physical activity and healthy eating in overweight and obese adults; systematic review and meta-regression analyses. International Journal of Behavioral Nutrition and Physical Activity.

[B55-behavsci-15-01448] Sequeira M., Pereira C., Alvarez M.-J. (2023). Promoting physical activity within breast cancer survivors using behaviour change techniques: N-of-1 randomized controlled trials. Health Promotion International.

[B56-behavsci-15-01448] Shea B. J., Reeves B. C., Wells G., Thuku M., Hamel C., Moran J., Moher D., Tugwell P., Welch V., Kristjansson E., Henry D. A. (2017). AMSTAR 2: A critical appraisal tool for systematic reviews that include randomised or non-randomised studies of healthcare interventions, or both. BMJ.

[B57-behavsci-15-01448] Sniehotta F. F., Scholz U., Schwarzer R. (2005). Bridging the intention–behaviour gap: Planning, self-efficacy, and action control in the adoption and maintenance of physical exercise. Psychology & Health.

[B58-behavsci-15-01448] Stevens M., Cruwys T., Murray K. (2020). Social support facilitates physical activity by reducing pain. British Journal of Health Psychology.

[B59-behavsci-15-01448] Tamuleviciute-Prasciene E., Beigiene A., Lukauskaite U., Gerulyte K., Kubilius R., Bjarnason-Wehrens B. (2022). Effectiveness of additional resistance and balance training and telephone support program in exercise-based cardiac rehabilitation on quality of life and physical activity: Randomized control trial. Clinical Rehabilitation.

[B60-behavsci-15-01448] Tang H., Zhang W., Weng Y., Zhang X., Shen H., Li X., Liu Y., Liu W., Xiao H., Jing H. (2025). Dietary self-management behaviour and associated factors among breast cancer patients receiving chemotherapy: A latent profile analysis. European Journal of Oncology Nursing.

[B61-behavsci-15-01448] Voss M. L., Brick R., Padgett L. S., Wechsler S., Joshi Y., Ammendolia Tome G., Arbid S., Campbell G., Campbell K. L., El Hassanieh D., Klein C., Lam A., Lyons K. D., Sabir A., Sleight A. G., Jones J. M. (2024). Behaviour change theory and behaviour change technique use in cancer rehabilitation interventions: A secondary analysis. European Journal of Physical and Rehabilitation Medicine.

[B62-behavsci-15-01448] Warburton D. E., Nicol C. W., Bredin S. S. (2006). Health benefits of physical activity: The evidence. CMAJ.

[B63-behavsci-15-01448] Webb J., Baker A., Palmer T., Hall A., Ahlquist A., Darlow J., Olaniyan V., Horlock R., Stewart D. (2022). The barriers and facilitators to physical activity in people with a musculoskeletal condition: A rapid review of reviews using the COM-B model to support intervention development. Public Health in Practice.

[B64-behavsci-15-01448] Willett M., Duda J., Fenton S., Gautrey C., Greig C., Rushton A. (2019). Effectiveness of behaviour change techniques in physiotherapy interventions to promote physical activity adherence in lower limb osteoarthritis patients: A systematic review. PLoS ONE.

[B65-behavsci-15-01448] World Health Organization (2023a). Noncommunicable diseases.

[B66-behavsci-15-01448] World Health Organization (2023b). Noncommunicable diseases: Key facts.

[B67-behavsci-15-01448] Zhang J., Brackbill D., Yang S., Becker J., Herbert N., Centola D. (2016). Support or competition? How online social networks increase physical activity: A randomized controlled trial. Preventive Medicine Reports.

[B68-behavsci-15-01448] Zhang X., Qiao X., Peng K., Gao S., Hao Y. (2024). Digital behaviour change interventions to reduce sedentary behaviour and promote physical activity in adults with diabetes: A systematic review and meta-analysis of randomized controlled trials. International Journal of Behavioral Medicine.

