# Supplementary material for: Application of Behaviour Change Techniques in Promoting Physical Activity Among Adults with Chronic Conditions: An Umbrella Review
Source: Behav Sci (Basel). 2025 Oct 24;15(11):1448. doi: 10.3390/bs15111448 (PMC12649445; doi:10.3390/bs15111448)
Supplement: Supplementary file 1 [file behavsci-15-01448-s001.zip › Supplemental material S2 Quality Appraisal.pdf]

Supplemental material 2: Quality Appraisal by Using AMSTAR 2

No	Study	Items of AMSTAR 2																
		1	2	3	4	5	6	7	8	9	10	11	12	13	14	15	16	Rate
1	Agirre-Elordui et al., 2024	Y	Y	Y	Y	Y	Y	PY	Y	Y	N	Y	Y	Y	N	Y	Y	High
2	Ashley et al., 2024	Y	Y	Y	Y	N	PY	Y	Y	Y	N	NA	NA	Y	N	NA	Y	Moderate
3	Carraça et al., 2021	Y	Y	Y	Y	Y	Y	PY	Y	Y	N	Y	Y	Y	Y	Y	Y	High
4	Cooper et al., 2023	Y	Y	Y	Y	Y	Y	PY	Y	Y	N	NA	NA	Y	PY	Y	Y	Moderate
5	de Leeuwerk et al., 2022	Y	Y	Y	Y	Y	PY	Y	Y	Y	N	Y	PY	Y	Y	Y	Y	High
6	Duff et al., 2017	Y	Y	Y	Y	Y	Y	Y	Y	Y	N	NA	NA	Y	Y	NA	Y	Moderate
7	Ester et al., 2021	Y	Y	Y	N	Y	Y	Y	Y	Y	N	NA	NA	Y	Y	NA	Y	LOW
8	Finne et al., 2018	Y	N	Y	PY	Y	Y	PY	Y	Y	N	Y	PY	Y	Y	PY	Y	LOW
9	Grimmett et al., 2019	Y	Y	Y	Y	Y	Y	PY	Y	Y	N	Y	PY	Y	Y	N	Y	LOW
10	Hailey et al., 2022	Y	Y	Y	Y	PY	Y	PY	Y	Y	N	NA	NA	Y	Y	NA	Y	Moderate
11	Lin et al., 2022	Y	Y	Y	Y	Y	Y	PY	Y	Y	N	NA	NA	Y	Y	NA	Y	Moderate
12	Marley et al., 2017	Y	Y	Y	Y	Y	Y	PY	Y	Y	N	Y	Y	Y	Y	N	Y	LOW
13	Mbous et al., 2020	Y	Y	Y	Y	Y	Y	PY	Y	Y	N	Y	Y	Y	Y	PY	Y	Moderate
14	Meade et al., 2019	Y	Y	Y	Y	Y	Y	PY	Y	Y	N	NA	NA	Y	Y	NA	Y	Moderate
15	Meyer-Schwickerath et al., 2022	Y	N	Y	Y	Y	Y	PY	Y	Y	N	NA	NA	Y	Y	PY	Y	LOW
16	Willett et al., 2019	Y	Y	Y	Y	Y	Y	PY	Y	Y	N	NA	NA	Y	Y	Y	Y	Moderate
17	Zhang et al., 2024	Y	Y	Y	PY	Y	Y	PY	Y	Y	N	Y	Y	Y	Y	Y	Y	High
18	Hallward et al., 2020	Y	Y	Y	Y	Y	Y	PY	Y	Y	N	NA	NA	Y	Y	NA	Y	Moderate

Notes: Items: 1.Research questions and inclusion criteria (PICO components); 2. Protocol and deviations; 3. Selection of study designs; 4. Literature search strategy; 5. Study selection in duplicate; 6. Data extraction in duplicate; 7. Exclusion of studies and justification; 8. Study descriptions; 9. Risk of bias assessment in included studies; 10. Funding sources for included studies; 11. Meta-analysis methods; 12. Impact of risk of bias on meta-analysis; 13. Risk of bias in interpretation; 14. Explanation of heterogeneity; 15. Publication bias investigation; 16. Conflict of interest disclosure.

AMSTAR 2 evaluates the methodological quality of systematic reviews by categorizing them into four levels. A review is considered **high** quality if it has no or only one non-critical weakness, providing an accurate and comprehensive summary of the available studies. Reviews with **moderate** quality have more than one non-critical weakness but no critical flaws, meaning they may still offer an accurate summary of the included studies. **Low** quality reviews contain one critical flaw, with or without non-critical weaknesses, and may not provide an accurate summary. Lastly, **critically low** reviews have more than one critical flaw and should not be relied upon for an accurate summary of the available studies. The green entries in the figure above represent key items in the evaluation. Additionally, the description of funding sources in 10 items is not used as a criterion for rating. The ratings are as follows: Y = Yes, PY = Partial Yes, N = No, NA = Not Applicable.
