# Supplementary material for: Application of Behaviour Change Techniques in Promoting Physical Activity Among Adults with Chronic Conditions: An Umbrella Review
Source: Behav Sci (Basel). 2025 Oct 24;15(11):1448. doi: 10.3390/bs15111448 (PMC12649445; doi:10.3390/bs15111448)
Supplement: Supplementary file 1 [file behavsci-15-01448-s001.zip › Supplementary Material S1 Searching Strategies.pdf]

Supplementary Material 1: Searching Strategies

Pubmed:
(("Behavior Change Techniques"[MeSH Terms] OR "BCT"[All Fields]) AND
("physical activity"[MeSH Terms] OR "exercise"[MeSH Terms]) AND
("chronic conditions"[All Fields] OR "diabetes"[MeSH Terms] OR "type 1 diabetes"[All Fields] OR "type 2 diabetes"[All Fields] OR "hypertension"[MeSH Terms] OR "obesity"[MeSH Terms] OR "cardiovascular diseases"[MeSH Terms] OR "coronary artery disease"[All Fields] OR "heart failure"[All Fields] OR "arthritis"[MeSH Terms] OR "rheumatoid arthritis"[All Fields] OR "osteoarthritis"[All Fields] OR "cancer"[MeSH Terms] OR "respiratory diseases"[MeSH Terms] OR "COPD"[All Fields] OR "stroke"[MeSH Terms] OR "chronic kidney disease"[All Fields] OR "CKD"[All Fields] OR "osteoporosis"[MeSH Terms] OR "depression"[MeSH Terms] OR "chronic pain"[All Fields] OR "fibromyalgia"[All Fields])) AND
("systematic review"[Publication Type] OR "meta-analysis"[Publication Type])

Embase:
('behavior change technique'/exp OR 'BCT') AND
('physical activity'/exp OR 'exercise'/exp) AND
('chronic disease'/exp OR 'diabetes mellitus'/exp OR 'type 1 diabetes'/exp OR 'type 2 diabetes'/exp OR 'hypertension'/exp OR 'obesity'/exp OR 'cardiovascular disease'/exp OR 'coronary artery disease'/exp OR 'heart failure'/exp OR 'arthritis'/exp OR 'rheumatoid arthritis'/exp OR 'osteoarthritis'/exp OR 'cancer'/exp OR 'chronic respiratory disease'/exp OR 'COPD'/exp OR 'stroke'/exp OR 'chronic kidney disease'/exp OR 'CKD'/exp OR 'osteoporosis'/exp OR 'depression'/exp OR 'chronic pain'/exp OR 'fibromyalgia'/exp) AND
('systematic review'/exp OR 'meta-analysis'/exp)

WOS:
TS=("Behavior Change Techniques" OR "BCT") AND
TS=("physical activity" OR "exercise") AND
TS=("chronic conditions" OR "diabetes" OR "type 1 diabetes" OR "type 2 diabetes" OR "hypertension" OR "obesity" OR "cardiovascular diseases" OR "coronary artery disease" OR "heart failure" OR "arthritis" OR "rheumatoid arthritis" OR "osteoarthritis" OR "cancer" OR "respiratory diseases" OR "COPD" OR "stroke" OR "chronic kidney disease" OR "CKD" OR "osteoporosis" OR "depression" OR "chronic pain" OR "fibromyalgia") AND
TS=("systematic review" OR "meta-analysis")

Cochrane:
[Behavior Change Techniques OR BCT] AND
[physical activity OR exercise] AND
[diabetes OR type 1 diabetes OR type 2 diabetes OR hypertension OR obesity OR cardiovascular diseases OR coronary artery disease OR heart failure OR arthritis OR rheumatoid arthritis OR osteoarthritis OR cancer OR respiratory diseases OR COPD OR stroke OR chronic kidney disease OR CKD OR osteoporosis OR depression OR chronic pain OR fibromyalgia] AND
[systematic review OR meta-analysis]

PsycINFO:

("Behavior Change Techniques" OR "BCT") AND
("physical activity" OR "exercise") AND
("chronic conditions" OR "diabetes" OR "type 1 diabetes" OR "type 2 diabetes" OR "hypertension" OR "obesity" OR "cardiovascular diseases" OR "coronary artery disease" OR "heart failure" OR "arthritis" OR "rheumatoid arthritis" OR "osteoarthritis" OR "cancer" OR "respiratory diseases" OR "COPD" OR "stroke" OR "chronic kidney disease" OR "CKD" OR "osteoporosis" OR "depression" OR "chronic pain" OR "fibromyalgia") AND
("systematic review" OR "meta-analysis")
