# Supplementary material for: Application of Behaviour Change Techniques in Promoting Physical Activity Among Adults with Chronic Conditions: An Umbrella Review
Source: Behav Sci (Basel). 2025 Oct 24;15(11):1448. doi: 10.3390/bs15111448 (PMC12649445; doi:10.3390/bs15111448)
Supplement: Supplementary file 1 [file behavsci-15-01448-s001.zip › Supplementary Material S4 Overlap Matirx.pdf]

Supplementary Material 4: Overlap Matrix

CCA=[(∑ni) −U]/[U×(k−1)]

CCA = (236-140)/140(9-1) = 96/1120 = 0.0857

		**Agirre-Elordui et al., 2024**	**Cooper et al., 2023**	**Ester et al., 2021**	**Finne et al., 2018**	**Grimmett et al., 2019**	**Hailey et al., 2022**	**Hallward et al., 2020**	**Mbous et al., 2020**	**Meyer-Schwickerath et al., 2022**	Unique Study
Total included studies		***5***	***20***	***71***	***42***	***27***	***28***	***18***	***11***	***14***	***236***
1	Abbott, 2017, DOI: 10.1188/17.CJON.618-626			✅							1
2	Adams, 2018, DOI: 10.1038/s41416-018-0044-7					✅					1
4	Alibhai, 2019, DOI: 10.1186/s12885-018-5189-5			✅							1
5	Allicock, 2021, DOI: 10.1007/s40615-020-00767-x		✅	✅							
6	Andersen, 2004, DOI: 10.1200/JCO.2004.06.030									✅	1

		**Agirre-Elordui et al., 2024**	**Cooper et al., 2023**	**Ester et al., 2021**	**Finne et al., 2018**	**Grimmett et al., 2019**	**Hailey et al., 2022**	**Hallward et al., 2018**	**Mbous et al., 2020**	**Meyer-Schwickerath et al., 2019**	Unique Study
7	Anderson, 2014, DOI: 10.1136/bmj.g1823								✅		1
8	Bade, 2018, DOI: 10.1177/1534735418781739			✅							1
9	Bantum, 2014, DOI: 10.2196/jmir.3020			✅	✅						
10	Basen-Engquist, 2006, DOI: 10.1016/j.pec.2006.02.006				✅		✅			✅	
11	Baumann, 2017, DOI: 10.1007/s00520-016-3490-x					✅	✅				
12	Befort, 2012, DOI: 10.1007/s10549-011-1922-3			✅							1
13	Belanger, 2014, DOI: 10.1089/jayao.2013.0021					✅					1
14	Bennett, 2007, DOI: 10.1097/00006199-200701000-00003				✅					✅	

		Agirre-Elordui et al., 2024	Cooper et al., 2023	Ester et al., 2021	Finne et al., 2018	Grimmett et al., 2019	Hailey et al., 2022	Hallward et al., 2018	Mbous et al., 2020	Meyer-Schwickerath et al., 2019	Unique Study
15	Berglund, 2007, DOI:10.1080/02841860600857326							✅			1
16	Beydoun, 2014, DOI: 10.1111/1754-9485.12115							✅			1
17	Bloom, 2008				✅						1
20	Brown, 2018,DOI: 10.1016/ j.clcc.2017.09.004								✅		1
21	Buscemi, 2020, DOI: 10.1007/ s10865-020-00136-3			✅							1
22	Cadmus, 2009, DOI: 10.1002/ pon.1525										1
23	Cairo, 2020, DOI: 10.17294/2330-0698.1733			✅							1
24	Campbell, 2009, DOI: 10.1007/ s12160-009-9140-5								✅		1
26	Campo, 2014, DOI: 10.1007/ s11764-013-0315-5							✅			1

		**Agirre-Elordui et al., 2024**	**Cooper et al., 2023**	**Ester et al., 2021**	**Finne et al., 2018**	**Grimmett et al., 2019**	**Hailey et al., 2022**	**Hallward et al., 2018**	**Mbous et al., 2020**	**Meyer-Schwickerath et al., 2019**	Unique Study
28	Carmack Taylor, 2004, DOI: 10.1016/j.cct.2004.03.001							✅			1
29	Carmack Taylor, 2006, DOI: 10.1002/pon.1023					✅				✅	
31	Chan, 2020, DOI: 10.2196/19238			✅							1
32	Chapman, 2018, DOI: 10.1111/bjhp.12313		✅	✅							
33	Cheong, 2018, DOI: 10.1016/j.clcc.2018.02.002			✅							1
34	Chow, 2021, DOI: 10.1007/s11764-020-00949-w			✅							1
35	Chung, 2019, DOI: 10.3389/fonc.2019.01505		✅	✅							
36	Courneya, 2016, DOI: 10.1158/1055-9965.EPI-15-1267	✅							✅		
37	Cox, 2017, DOI: 10.2196/cancer.7166			✅							1

		**Agirre-Elordui et al., 2024**	**Cooper et al., 2023**	**Ester et al., 2021**	**Finne et al., 2018**	**Grimmett et al., 2019**	**Hailey et al., 2022**	**Hallward et al., 2018**	**Mbous et al., 2020**	**Meyer-Schwickerath et al., 2019**	Unique Study
38	Culos-Reed, 2007, DOI: 10.1123/jsep.29.1.118							✅			1
39	Culos-Reed, 2010, DOI: 10.1007/s00520-009-0694-3				✅			✅			
40	Daley, 2007, DOI: 10.1200/JCO.2006.09.5083						✅				1
41	De Luca, 2016, DOI: 10.1016/j.puhe.2016.03.028						✅				1
42	Demark-Wahnefried (Main outcomes), 2007, DOI: 10.1200/JCO.2007.10.7094				✅						1
43	Demark-Wahnefried, 2003, DOI: 10.1249/01.MSS.0000053704.28156.0F				✅						1
44	Demark-Wahnefried, 2012, DOI: 10.1200/JCO.2011.40.0895				✅	✅					
45	Dhillon, 2017, DOI: 10.1093/annonc/mdx205					✅					1

		Agirre-Elordui et al., 2024	Cooper et al., 2023	Ester et al., 2021	Finne et al., 2018	Grimmett et al., 2019	Hailey et al., 2022	Hallward et al., 2018	Mbous et al., 2020	Meyer-Schwickerath et al., 2019	Unique Study
46	Di Blasio, 2022, DOI: 10.1080/01621424.2021.1984362		✅								1
48	Edbrooke, 2019, DOI: 10.3390/jcm8091288			✅							1
49	Fazzino, 2017, DOI: 10.1002/oby.22007			✅							1
50	Ferrante, 2020, DOI: 10.1093/tbm/iby124		✅								1
51	Forbes, 2015, DOI: 10.2196/cancer.4586			✅							1
52	Frensham, 2018, DOI: 10.3390/ijerph15102081			✅							1
53	Frensham, 2020, DOI: 10.1097/NCC.0000000000000649			✅							1
54	Galvao, 2009, DOI: 10.1186/1471-2407-9-419							✅			1

		Agirre - Elordui et al., 2024	Cooper et al., 2023	Ester et al., 2021	Finne et al., 2018	Grimmett et al., 2019	Hailey et al., 2022	Hallward et al., 2018	Mbous et al., 2020	Meyer-Schwickerath et al., 2019	Unique Study
55	Galvao, 2014, DOI: 10.1016/ j.eururo.2013.09.041							✅			1
56	Galvao, 2018, DOI: 10.1002/ pon.4495					✅					1
57	Gehring, 2018, DOI: 10.1177/0269215517728326			✅							1
58	Gell, 2017, DOI: 10.1007/ s00520-016-3523-5			✅							1
59	Gell, 2020, DOI: 10.1007/ s11764-019-00831-4			✅							1
60	Gokal, 2016, DOI: 10.1007/ s00520-015-2884-5			✅							1
61	Golsteijn, 2018, DOI: 10.1186/ s12966-018-0734-9			✅							1
62	Gomersall, 2019, DOI: 10.1186/ s12889-019-6767-4			✅							1
63	Greenlee, 2013, DOI: 10.1002/ oby.20245						✅				1

		Agirre-Elordui et al., 2024	Cooper et al., 2023	Ester et al., 2021	Finne et al., 2018	Grimmett et al., 2019	Hailey et al., 2022	Hallward et al., 2018	Mbous et al., 2020	Meyer-Schwickerath et al., 2019	Unique Study
64	Groen, 2017, DOI: 10.2196/cancer.7443			✅							1
65	Haggerty, 2017, DOI: 10.1002/oby.22021			✅							1
66	Harrigan, 2016, DOI: 10.1200/JCO.2015.61.6375						✅			✅	
67	Hartman, 2018, DOI: 10.2196/mhealth.8503			✅							1
68	Hatchett, 2013, DOI: 10.1002/pon.3082		✅	✅	✅		✅				
69	Hawkes, 2013, DOI: 10.1200/JCO.2012.45.5873	✅				✅			✅		
71	Hebert, 2012, DOI: 10.1016/j.canep.2011.09.008				✅						1
72	Hirschey, 2018						✅				1
73	Holtdirk, 2021, DOI: 10.1371/journal.pone.0251276		✅								1

		Agirre-Elordui et al., 2024	Cooper et al., 2023	Ester et al., 2021	Finne et al., 2018	Grimmett et al., 2019	Hailey et al., 2022	Hallward et al., 2018	Mbous et al., 2020	Meyer-Schwickerath et al., 2019	Unique Study
74	Hong, 2015, DOI: 10.2196/ cancer.4389			✅							1
75	Hoybye, 2008, DOI: 10.1080/02841860701418846				✅						1
76	Ibfelt, 2011, DOI: 10.3109/0284186X.2010.531761				✅						1
77	Irwin, 2008, DOI: 10.1002/ cncr.23446				✅		✅				
79	James, 2011, DOI: 10.1186/1471-2458-11-236				✅						1
80	James, 2015, DOI: 10.1186/ s12885-015-1775-y				✅	✅					
81	Javaheri, 2015, DOI: 10.3138/ ptc.2014-24O			✅							1
83	Kanera, 2016, DOI: 10.1007/ s11764-016-0535-6			✅							1
84	Kanera, 2017, DOI: 10.1186/ s12966-017-0474-2			✅		✅					

		Agirre-Elordui et al., 2024	Cooper et al., 2023	Ester et al., 2021	Finne et al., 2018	Grimmett et al., 2019	Hailey et al., 2022	Hallward et al., 2018	Mbous et al., 2020	Meyer-Schwickerath et al., 2019	Unique Study
85	Kenfield, 2019, DOI: 10.1016/j.eururo.2018.12.040			✅							1
88	Kim, 2011, DOI: 10.1188/11.ONF.E97-E106				✅		✅				
89	Kim, 2020, DOI: 10.1007/s00520-020-05363-7		✅								1
90	Koutoukidis, 2019, DOI: 10.1136/ijgc-2018-000039									✅	1
91	Kuijpers, 2016, DOI: 10.2196/cancer.5456		✅								1
92	Lahart, 2016, DOI: 10.1186/s12885-016-2258-5				✅		✅				
93	Lahart, 2018, DOI: 10.1002/14651858.CD011292.pub2						✅				1
94	Leclerc, 2018, DOI: 10.1016/j.clbc.2018.05.004					✅	✅				
95	Lee (CF), 2018, DOI: 10.1038/s41598-018-24042-6					✅					1

		**Agirre-Elordui et al., 2024**	**Cooper et al., 2023**	**Ester et al., 2021**	**Finne et al., 2018**	**Grimmett et al., 2019**	**Hailey et al., 2022**	**Hallward et al., 2018**	**Mbous et al., 2020**	**Meyer-Schwickerath et al., 2019**	Unique Study
96	Lee (MK), 2018, DOI: 10.1371/journal.pone.0196220									✅	1
97	Lee, 2013, DOI: 10.1007/s00520-013-1822-7								✅		1
98	Lee, 2014, DOI: 10.1016/j.ijnurstu.2014.04.012		✅								1
99	Lee, 2018, DOI: 10.1038/s41598-018-24042-6	✅							✅		
100	Ligibel, 2012, DOI: 10.1007/s10549-011-1882-7				✅						1
101	Livingston, 2011, DOI: 10.1186/1471-2407-11-237				✅			✅			
102	Livingston, 2015, DOI: 10.1002/cncr.29385				✅			✅			
103	Lynch, 2019 (Maintenance), DOI: 10.1002/cncr.32142			✅							1
104	Lynch, 2019 (RCT), DOI: 10.1002/cncr.32143		✅	✅			✅				

		**Agirre-Elordui et al., 2024**	**Cooper et al., 2023**	**Ester et al., 2021**	**Finne et al., 2018**	**Grimmett et al., 2019**	**Hailey et al., 2022**	**Hallward et al., 2018**	**Mbous et al., 2020**	**Meyer-Schwickerath et al., 2019**	Unique Study
105	MacDonald, 2020, DOI: 10.3390/jcm9103092			✅							1
106	Matthews, 2007, DOI: 10.1007/s00520-006-0122-x				✅		✅				
107	Maxwell-Smith, 2019, DOI: 10.1002/pon.5090			✅						✅	
108	Mayer, 2018, DOI: 10.1007/s11764-017-0647-7	✅		✅		✅			✅		
109	Mayo, 2014, DOI: 10.1177/0269215514536209			✅							1
110	McCarroll, 2015, DOI: 10.1016/j.ygyno.2014.12.025			✅							1
111	McGowan, 2013, DOI: 10.1007/s12160-013-9519-1							✅			1
113	McNeil, 2019, DOI: 10.1249/MSS.0000000000001890			✅			✅				
114	Mefferd, 2007, DOI: 10.1007/s10549-006-9410-x									✅	1

		**Agirre-Elordui et al., 2024**	**Cooper et al., 2023**	**Ester et al., 2021**	**Finne et al., 2018**	**Grimmett et al., 2019**	**Hailey et al., 2022**	**Hallward et al., 2018**	**Mbous et al., 2020**	**Meyer-Schwickerath et al., 2019**	Unique Study
116	Morey, 2009, DOI: 10.1001/jama.2009.643				✅						1
118	Mutrie, 2012, DOI: 10.1007/s11764-012-0233-y					✅					1
119	Naito, 2019, DOI: 10.1002/jcsm.12351			✅							1
120	Napoles, 2019, DOI: 10.2196/13543			✅							1
121	Nyrop, 2017, DOI: 10.1634/theoncologist.2017-0174					✅					1
122	Nyrop, 2018, DOI: 10.1007/s10549-017-4565-1			✅							1
123	O'Neill, 2018, DOI: 10.1097/SLA.0000000000002895					✅					1
124	Ochi, 2022, DOI: 10.1136/bmjsp-care-2021-003141		✅								1

		**Agirre-Elordui et al., 2024**	**Cooper et al., 2023**	**Ester et al., 2021**	**Finne et al., 2018**	**Grimmett et al., 2019**	**Hailey et al., 2022**	**Hallward et al., 2018**	**Mbous et al., 2020**	**Meyer-Schwickerath et al., 2019**	Unique Study
125	Ormel, 2018, DOI: 10.1007/s00520-018-4263-5			✅							1
126	Ottenbacher, 2012, DOI: 10.1007/s00520-011-1370-y				✅	✅					
127	Painter, 2008, DOI: 10.1007/s12160-008-9042-y								✅		1
128	Park (JH), 2015, DOI: 10.1002/cncr.29400			✅			✅			✅	
129	Park (S), 2020, DOI: 10.5993/AJHB.44.1.10			✅							1
130	Park (SW), 2019, DOI: 10.1007/s00520-018-4427-3			✅							1
131	Park, 2015, DOI: 10.1002/cncr.29400				✅						1
132	Paxton, 2017, DOI: 10.2196/cancer.7495			✅							1
134	Phillips, 2022, DOI: 10.1002/cncr.34012		✅								1

		**Agirre-Elordui et al., 2024**	**Cooper et al., 2023**	**Ester et al., 2021**	**Finne et al., 2018**	**Grimmett et al., 2019**	**Hailey et al., 2022**	**Hallward et al., 2018**	**Mbous et al., 2020**	**Meyer-Schwickerath et al., 2019**	Unique Study
136	Pinto, 2005, DOI: 10.1200/ JCO.2005.03.080				✅		✅				
137	Pinto, 2008, DOI: 10.1007/ s00520-008-0434-0				✅	✅					
138	Pinto, 2013, DOI: 10.1002/ pon.2047	✅							✅		
139	Pinto, 2013, DOI: 10.1037/ a0029886				✅						
140	Pinto, 2013, DOI::10.1002/ pon.3272						✅				1
141	Pinto, 2015, DOI: 10.1037/ hea0000120						✅				1
142	Pinto, DOI: 10.1002/pon.2047					✅					1
143	Pinto, DOI: 10.1037/a0029886					✅					1
144	Pinto, DOI: 10.1037/hea0000120					✅					1

		Agirre-Elordui et al., 2024	Cooper et al., 2023	Ester et al., 2021	Finne et al., 2018	Grimmett et al., 2019	Hailey et al., 2022	Hallward et al., 2018	Mbous et al., 2020	Meyer-Schwickerath et al., 2019	Unique Study
145	Pope (Z), 2019, DOI: 10.1093/tbm/iby002			✅							1
146	Pope (ZC), 2018, DOI: 10.3390/jcm7060140		✅	✅							
147	Pope, 2019, DOI: 10.1093/tbm/iby002		✅								1
148	Purcell, 2011, DOI: 10.1007/s00520-010-0970-2									✅	1
149	Puszkiewicz, 2016, DOI: 10.2196/cancer.5380			✅							1
150	Quintiliani, 2016		✅								1
151	Rabin, 2011, DOI: 10.1089/jayao.2011.0040			✅							1
152	Rabin, 2016, DOI: 10.1089/jayao.2015.0033				✅						1
153	Reif, 2010, DOI: 10.1007/s00063-010-1132-5 *(German article)*				✅						1

		**Agirre-Elordui et al., 2024**	**Cooper et al., 2023**	**Ester et al., 2021**	**Finne et al., 2018**	**Grimmett et al., 2019**	**Hailey et al., 2022**	**Hallward et al., 2018**	**Mbous et al., 2020**	**Meyer-Schwickerath et al., 2019**	Unique Study
154	Reif, 2013, DOI: 10.1016/j.ejon.2012.07.002				✅					✅	
155	Robertson, 2020, DOI: 10.2196/18364			✅							1
156	Rogers, 2009, DOI: 10.1158/1055-9965.EPI-08-1045					✅					1
157	Rogers, 2009, DOI: 10.1249/MSS.0b013e31818e0e1b						✅				1
158	Rogers, 2012, DOI: 10.1016/j.cct.2011.09.004				✅						1
159	Rogers, 2014, DOI: 10.1249/MSS.0000000000000210						✅				1
160	Rogers, 2015, DOI: 10.1007/s10549-014-3216-z				✅	✅	✅				
161	Saarto, 2012						✅				1

		Agirre-Elordui et al., 2024	Cooper et al., 2023	Ester et al., 2021	Finne et al., 2018	Grimmett et al., 2019	Hailey et al., 2022	Hallward et al., 2018	Mbous et al., 2020	Meyer-Schwickerath et al., 2019	Unique Study
162	Sajid, 2016, DOI: 10.1016/j.jgo.2016.02.002							✅			1
163	Sandler, 2017, DOI: 10.1016/j.jpainsymman.2017.03.015					✅					1
164	Santa Mina, 2012, DOI: 10.4236/jct.2012.32020							✅			1
165	Santa Mina, 2013, DOI: 10.1123/japa.21.4.455							✅			1
167	Serda, 2010							✅			1
168	Shang, 2012, DOI: 10.1097/NCC.0b013e318236a3b3			✅							1
169	Sheean, 2021, DOI: 10.1007/s10549-021-06163-1		✅								1
170	Sheppard, 2016, DOI: 10.1016/j.cct.2015.12.005				✅		✅				
171	Short (CE), 2017, DOI: 10.1007/s11764-016-0565-0			✅							1

		**Agirre-Elordui et al., 2024**	**Cooper et al., 2023**	**Ester et al., 2021**	**Finne et al., 2018**	**Grimmett et al., 2019**	**Hailey et al., 2022**	**Hallward et al., 2018**	**Mbous et al., 2020**	**Meyer-Schwickerath et al., 2019**	Unique Study
172	Short (CE), 2018, DOI: 10.1186/s12913-017-2818-7			✅							1
173	Short, 2012, DOI: 10.1186/1471-2407-12-172				✅						1
174	Short, 2013, DOI: 10.1186/1479-5868-10-124				✅						1
175	Short, 2015, DOI: 10.1002/pon.3639				✅		✅				
176	Singh, 2020, DOI: 10.1016/j.jsams.2019.09.019		✅	✅							
177	Snyder, 2009, DOI: 10.1002/pon.1491				✅						1
178	Spark, 2015, DOI: 10.2196/mhealth.4114			✅							1
179	Stolley, 2017, DOI: 10.1200/JCO.2016.71.9856					✅	✅				
180	Sturgeon, 2017, DOI: 10.1007/s11764-016-0582-z		✅								1

		**Agirre-Elordui et al., 2024**	**Cooper et al., 2023**	**Ester et al., 2021**	**Finne et al., 2018**	**Grimmett et al., 2019**	**Hailey et al., 2022**	**Hallward et al., 2018**	**Mbous et al., 2020**	**Meyer-Schwickerath et al., 2019**	Unique Study
181	Taylor, 2004, DOI: 10.1016/ j.cct.2004.03.001				✅						1
182	Taylor, 2006, DOI: 10.1002/ pon.1023				✅						1
184	Trinh, 2018, DOI: 10.1186/ s12966-018-0686-0			✅							1
185	Truong, 2011, DOI: 10.1097/ COC.0b013e3181e841ec							✅			1
186	Uhm, 2017, DOI: 10.1007/ s10549-016-4065-8		✅	✅							
187	Vallance 2008, DOI: 10.1177/1090198106287693				✅						1
188	Vallance, 2007, DOI: 10.1200/ JCO.2006.07.9988				✅		✅				
189	Vallance, 2008, DOI: 10.1249/ mss.0b013e3181586b41					✅					1

		**Agirre-Elordui et al., 2024**	**Cooper et al., 2023**	**Ester et al., 2021**	**Finne et al., 2018**	**Grimmett et al., 2019**	**Hailey et al., 2022**	**Hallward et al., 2018**	**Mbous et al., 2020**	**Meyer-Schwickerath et al., 2019**	Unique Study
190	Valle, 2013, DOI: 10.1007/s11764-013-0279-5			✅							1
191	Van Blarigan, 2019, DOI: 10.1186/s12885-019-5427-5			✅							1
192	van Waart, 2018, DOI: 10.1007/s00384-017-2921-6								✅		1
193	Villaron, 2018, DOI: 10.1007/s00520-018-4191-4			✅							1
194	von Gruenigen, 2008, DOI: 10.1016/j.ygyno.2007.12.026									✅	1
195	von Gruenigen, 2012, DOI: 10.1016/j.ygyno.2012.03.042				✅	✅				✅	
196	Webb (1), 2019, Public Health **(171)**, DOI: 10.1016/j.puhe.2019.04.006			✅							1
197	Webb (2), 2019, Public Health **(175)**, DOI: 10.1016/j.puhe.2019.06.013			✅							1

		**Agirre-Elordui et al., 2024**	**Cooper et al., 2023**	**Ester et al., 2021**	**Finne et al., 2018**	**Grimmett et al., 2019**	**Hailey et al., 2022**	**Hallward et al., 2018**	**Mbous et al., 2020**	**Meyer-Schwickerath et al., 2019**	Unique Study
198	Weiner, 2019, DOI: 10.1007/s11764-019-00768-8			✅							1
199	Wilson, 2005, *Prev Chronic Dis*, 2(2):A16 (DOI not explicitly listed in data)			✅							1
200	Witlox, 2018, DOI: 10.1186/s12916-018-1075-x					✅					1
201	Yun, 2020, DOI: 10.1002/pon.5394			✅							1
202	Zahavich, 2013, DOI: 10.1177/1534735412473640							✅			1
203	Zhang, 2017, DOI: 10.1188/17.ONF.366-374			✅							1
**Total unique studies**											**140**
