# Supplementary material for: Application of Behaviour Change Techniques in Promoting Physical Activity Among Adults with Chronic Conditions: An Umbrella Review
Source: Behav Sci (Basel). 2025 Oct 24;15(11):1448. doi: 10.3390/bs15111448 (PMC12649445; doi:10.3390/bs15111448)
Supplement: Supplementary file 1 [file behavsci-15-01448-s001.zip › Supplimental Material S3 Characteristis of Included Studies.pdf]

Supplimental material 3. Characteristics of Included Studies

No	**Basic Characteristics** (*Author, year, article title, Review category, Number of studies*)	**Population AND Sample size**	**Intervention Delivery mode/technique**	**PA Intervention effectiveness**	**BCT labels and frequency of BCTs**	**Effectiveness of BCTs or components**	**Evidence quality**
**1**	Zhang et al., 2024<br><br>Digital Behavior Change Interventions to Reduce Sedentary Behavior and Promote Physical Activity in Adults with Diabetes: A Systematic Review and Meta-Analysis of Randomized Controlled Trials<br><br>SR + MA<br><br>13 studies<br>All RCTs	Adults with type 2 diabetes<br><br>N = 980 (average age ranged from 50 to 67.3 years)	DBCIs, including text messages, websites, apps, activity trackers, and games<br>Personalized features such as feedback, goal setting, and tailored advice<br><br>Duration: 5 weeks to 6 months (mean = 15weeks)	Steps: SMD = 0.56 (95% CI: 0.08, 1.05)<br>LPA: SMD = 0.13 (95% CI: −0.06, 0.33)<br>MVPA: SMD = 0.14 (95% CI: −0.03, 0.32)	Total 29 BCTs Used:<br>1.1; 1.2; 1.3; 1.4; 1.5; 1.6; 1.7; 2.2; 2.3; 2.4; 3.1; 3.2; 3.3; 4.1; 4.3; 5.1 ; 5.2; 5.4; 6.1; 7.1; 8.3; 8.4; 8.7; 9.2; 10.3; 13.2; 13.4; 15.1; 15.4<br><br>The average number of BCTs reported in a study was 9.8 (range 4–24; median 4), and the average number of BCT clusters was 5.5 (range 3–10; median 6)<br><br>Most used BCTs<br>2.2 (n = 12); 1.1 (n = 11); 1.4 (n = 10); 5.1 (n = 10); 1.2 (n = 9); 1.5 (n = 9); 2.3 (n = 9); 7.1 (n = 9)	Findings:<br>DBCIs with more than 10 BCTs showed significant improvements in steps, LPA, and MVPA<br><br>DBCIs of moderate and long durations, involving over 4 BCT clusters or having a face-to-face component, led to significant increases in steps<br><br>Studies with at least 2 DBCI components were effective in improving steps, increasing LPA and MVPA, and reducing sedentary time	ROB: Blinding was a challenge due to the nature of DBCIs, leading to performance bias of step count and MVPA<br><br>Sensitivity analyses confirmed the robustness of results in most outcomes
**2**	Carraça et al., 2021<br><br>Effective behavior change techniques to promote physical activity in adults with overweight or obesity: A systematic review and meta-analysis<br><br>SR + MA<br><br>62 studies<br>59 RCTs and 3 non-RCTs	Adults with overweight or obesity (BMI ≥ 25 kg/m² or BMI ≥ 30 kg/m²)<br>Includes comorbidities like metabolic syndrome, type 2 diabetes, hypertension, and dyslipidemia in some studies<br><br>N = 12,854 (digital: 6240; face-to-face: 6614)	Main Intervention: BCTs applied via digital or face-to-face delivery to promote PA<br><br>Duration: 2 to 78 weeks<br><br>Follow-up (23 studies, 37%): median 39 weeks	Digital: SMD = 0.42 (95% CI: 0.28–0.57), I^2 = 92%<br>Face-to-face: SMD = 0.78 (95% CI: 0.51–1.01), I^2 = 96%	Total 54 BCTs Used:<br>1.1, 1.2, 1.3, 1.4, 1.5, 1.6, 1.8, 1.9, 2.1, 2.2, 2.3, 2.4, 2.5, 2.7, 3.1, 3.3, 4.1, 4.2, 5.1, 5.4, 5.6, 6.1, 6.2, 6.3, 7.1, 8.1, 8.7, 9.1, 9.2, 9.3, 10.1, 10.2, 10.3, 10.4, 10.5, 10.6, 10.7, 10.8, 10.9, 11.2, 12.2, 12.3, 12.4, 12.5, 13.1, 13.2, 13.4, 13.5, 14.1, 14.4, 15.1, 15.3, 15.4, 16.3<br><br>Digital interventions: 1–21 techniques (median: 8)<br>Face-to-face interventions: 1–26 techniques (median: 6)<br><br>Most Common BCTs:<br>1.1;1.2; 2.2; 2.3; 2.4; 3.1; 4.1; 5.1; 7.1; 8.1; 8.7; 9.1	Five BCTs showed significant moderator effects on PA in digital interventions: 1.1, 1.3, 8.7, 10.5 for positive effects and 2.3 for negative effects<br><br>One BCT showed significant positive moderator effects on PA in face-to-face interventions: 8.1<br><br>The total number of techniques used did not show significant moderating effects	Quality Ratings:<br>Good: 15 studies.<br>Fair: 26 studies.<br>Poor: 21 studies.<br>Issues: High dropout rates (>20%), limited use of objective PA measures, and lack of intent-to-treat analysis in several studies

No	Basic Characteristics (*Author, year, article title, Review category, Number of studies)*	Population AND Sample size	Intervention Delivery mode/technique	PA Intervention effectiveness	BCT labels and frequency of BCTs	Effectiveness of BCTs or components	Evidence quality
**3**	Ashley et al., 2024<br><br>Identifying the active ingredients of training interventions for healthcare professionals to promote and support increased levels of physical activity in adults with heart failure: A systematic review<br><br>SR<br><br>10 studies<br>All RCTs	Adults with heart failure receiving interventions focused on promoting PA<br><br>N = 1,414	Training modes included in-person sessions, virtual formats, and use of educational materials/manuals<br><br>Duration: 1 to 4 days	Four studies: Statistically significant PA improvements.<br>Five studies: Within-group PA improvement, no between-group difference<br>Five studies: No significant changes in PA outcomes.<br>Variability: Differences in intervention design and reporting	7 distinct BCTs identified across all interventions:<br>1.2 (n = 4); 4.1 (n = 3); 8.1 (n = 3); 2.2 (n = 2); 6.1 (n = 2); 1.5 (n= 1); 5.1 (n = 1)<br><br>The median number of BCTs used within training interventions was 1.5 (IQR = 2)	4.1 was frequently associated with improved PA outcomes	ROB: 6 studies rated as low risk; 4 studies rated as having some concerns due to issues such as lack of blinding or inadequate reporting of randomization procedures
**4**	Duff et al., 2017<br><br>Behavior Change Techniques in Physical Activity eHealth Interventions for People With Cardiovascular Disease: Systematic Review<br><br>SR<br><br>23 studies<br>All RCTs	Adults aged 18 years and older clinically diagnosed with cardiovascular disease (CVD).<br>Specific Conditions: Included individuals with myocardial infarction, heart failure, coronary heart disease (CHD), among others<br><br>N = 3,633	BCTs applied via digital platforms to promote PA.<br>Delivery Methods: Technological Components: Included Internet-based communications, mobile phone interventions, telehealth devices, and virtual reality tools<br><br>Duration: 3 weeks to 16 months (mean = 4.5 months)	8 out of 15 interventions: Significant PA improvement vs. control<br>eHealth: As effective or superior to standard cardiac rehab for PA	Total 30 BCTs Identified:<br>1.1, 1.2, 1.3, 1.4, 1.5, 1.7, 1.8, 2.1, 2.2, 2.3, 2.4, 2.5, 2.6, 2.7, 3.1, 3.2, 3.3, 4.1, 5.1, 6.1, 6.2, 7.1, 8.7, 9.1, 10.3, 10.4, 11.1, 11.2, 12.1, 12.5<br><br>The average number of BCTs used across the 23 studies was 7.2 (range 1-19)<br><br>Most Frequently Used BCTs:<br>5.1 (n=18, 78%)<br>1.1 (n=17, 74%)<br>2.3 (n=11, 48%)<br>3.2 (n=11, 48%)	BCTs Linked to Improved PA Outcomes<br>1.1 and 5.1 were the most frequently used in successful interventions<br>2.2 and 4.1 were also common in effective interventions<br>Additional: 2.3, 3.2, 3.1, 9.1, 1.2, 1.5, 10.4, 11.1<br><br>Non-Effective BCTs<br>3.2 and 5.1 in self-reported studies<br>1.1 and 2.2 in objectively measured studies<br>Both effective and ineffective interventions used an average of seven BCTs<br>No single BCT or combination was universally effective across all studies	Variable methodological quality (quasi-RCTs, narrative synthesis)<br><br>Certainty of Evidence Overall moderate to low

No	Basic Characteristics (*Author, year, article title, Review category, Number of studies)*	Population AND Sample size	Intervention Delivery mode/technique	PA Intervention effectiveness	BCT labels and frequency of BCTs	Effectiveness of BCTs or components	Evidence quality
**5**	Marley et al., 2017<br><br>The effectiveness of interventions aimed at increasing physical activity in adults with persistent musculoskeletal pain: a systematic review and meta-analysis<br><br>SR + MA<br><br>19 studies<br>19 RCTs<br>1 Non-RCT	Adults (≥18 years) with persistent musculoskeletal pain (PMP) in the axial skeleton or large peripheral joints (e.g., low back pain, osteoarthritis) for ≥3 months<br><br>N = 3,441	Delivery modes varied: Home-based; Center-based or inpatient; Web-based or automated; Combinations of above modes<br><br>Duration: 4 weeks to 12 months<br><br>Follow-up: longest 18 months	Subjective measures of PA (15 studies pooled):<br>Short term (≤ 12 weeks): No statistically significant effect (very low-quality evidence).<br>Medium term (3–6 months): SMD = 0.25 (95% CI: 0.01, 0.48)<br>Long term (> 6 months): SMD = 0.21 (95% CI: 0.08, 0.33).	Total BCTs identified: 60 BCTs across 20 studies.<br><br>The mean number of BCTs coded was eight (range 0–16)<br><br>Most frequent BCTs:<br>1.1(n = 13); 4.1(n = 13); 8.1(n = 11); 2.3(n = 13)	While a precise quantitative moderator analysis was not feasible, descriptive observations suggest: 1.1, 4.1, and 2.3 may be linked to more positive outcomes.	ROB: Many studies did not blind participants or providers (common in PA interventions).<br>Several were not explicitly powered for PA outcomes.<br><br>GRADE ratings ranged from moderate to very low.

No	Basic Characteristics (*Author, year, article title, Review category, Number of studies)*	Population AND Sample size	Intervention Delivery mode/technique	PA Intervention effectiveness	BCT labels and frequency of BCTs	Effectiveness of BCTs or components	Evidence quality
**6**	Meade et al., 2019<br><br>Behaviour change techniques associated with adherence to prescribed exercise in patients with persistent musculoskeletal pain: Systematic review<br><br>SR<br><br>8 studies<br>All RCTs	Adults aged 18–65 years with persistent musculoskeletal pain (PMSK) of ≥3 months' duration.<br>PMSK conditions included low back pain, fibromyalgia, whiplash disorders, etc.<br><br>N = 1,018	Delivery varied:<br>Some interventions were delivered face to face by a physiotherapist, psychologist, exercise specialist, or physician.<br>Settings included clinics or community health centres.<br>Some used group exercise or progressive, graded exercise approaches.<br><br>Duration: only reported the session duration from 30 minutes to over 2 hours	5 of 8 RCTs: Significant improvement in exercise adherence favouring intervention	Total 30 distinct BCTs identified: 1.1; 1.2; 1.3; 1.4; 1.5; 1.6; 1.7; 1.8; 1.9; 2.1; 2.2; 2.3; 2.6; 3.1; 4.1; 5.1; 5.4; 6.1; 7.1; 8.1; 8.6; 8.7; 9.1; 9.3; 10.3; 10.4; 10.11; 12.5; 12.6; 13.2<br><br>The number of BCTs varied from three to eleven (median 7)<br><br>Most common across interventions:<br>4.1(n = 7); 6.1(n = 4); 8.1(n = 5)	Using a Cochrane Back Group–style rating, moderate evidence emerged that the following BCTs were associated with improved adherence: 3.1; 1.1; 4.1; 6.1; 8.1<br><br>Interventions using ≤7 BCTs and ensuring these BCTs were unique to the intervention arm (i.e., not also used in the control) appeared most effective	ROB: 5 studies rated low risk; 3 studies rated high risk<br><br>Common issues included lack of blinding and incomplete reporting of intervention fidelity

No	Basic Characteristics (*Author, year, article title, Review category, Number of studies)*	Population AND Sample size	Intervention Delivery mode/technique	PA Intervention effectiveness	BCT labels and frequency of BCTs	Effectiveness of BCTs or components	Evidence quality
**7**	Willett et al., 2019<br><br>Effectiveness of behaviour change techniques in physiotherapy interventions to promote physical activity adherence in lower limb osteoarthritis patients: A systematic review<br><br>SR<br><br>24 studies<br>All RCTs	Adults (≥18 years) diagnosed with lower-limb osteoarthritis (hip and/or knee OA)<br><br>N = 2,366	Mode of Delivery: Individual (1-to-1) physiotherapy interventions incorporating at least 1 distinct BCT to promote home- or community-based PA adherence<br>Majority used in-person sessions plus follow-up phone calls, diaries, or other forms of remote support<br><br>Duration: NA	Most interventions showed positive short-term improvements in PA adherence (up to 3 months), though fewer sustained changes at medium-term (6 months) or long-term (≥12 months)	Total 26 Distinct BCTs Identified: 1.1; 1.2; 1.3; 1.4; 1.5; 1.8; 2.1; 2.2; 2.3; 2.7; 3.1; 4.1; 4.2; 5.1; 5.3; 6.1; 8.1; 8.6; 8.7; 9.1; 10.3; 11.2; 12.4; 12.5; 12.6; 15.4<br><br>11.2 (2.9) BCTs (ranged 5 to 20) average study were coded<br><br>Most Common BCTs (across interventions):<br>8.6 (100%); 9.1 (100%); 12.6 (100%); 1.4 (94%); 4.1 (94%); 8.1 (87%); 1.1 (71%); 2.3 (58%); 8.7 (52%)	Key BCTs with Higher “Effectiveness Ratios” (≥ 50% in more than one time point):<br>1.1; 1.8; 2.3; 3.1; 10.3	ROB: 5 studies rated low risk; 7 moderate and 12 high risk<br><br>Common issues included lack of participant blinding, incomplete outcome data, and selective reporting

No	**Basic Characteristics** (*Author, year, article title, Review category, Number of studies)*	**Population AND Sample size**	**Intervention Delivery mode/technique**	**PA Intervention effectiveness**	**BCT labels and frequency of BCTs**	**Effectiveness of BCTs or components**	**Evidence quality**
**8**	de Leeuwerk et al., 2022<br><br>The effectiveness of physical activity interventions using activity trackers during or after inpatient care: a systematic review and meta-analysis of randomized controlled trials<br><br>SR + MA<br><br>21 studies<br>All RCTs	Adults during or within three months after hospitalization or inpatient rehabilitation<br><br>N = 2,355	Behavior change strategies via activity trackers (e.g., accelerometer, pedometer) with feedback, possibly including coaching, goal setting, and action planning, based on a theoretical model (e.g., Social Cognitive Theory), delivered during or after inpatient care<br><br>Duration: 5 days to 12 months<br><br>Follow-up (only one study): 6 months	SMD = 0.34 (95% CI: 0.12, 0.56)	20 BCTs identified in the intervention groups:<br>1.1; 1.2; 1.3; 1.4; 1.5; 1.6; 2.2; 2.3; 2.4; 2.7; 3.1; 4.1; 5.1; 5.3; 6.2; 7.1; 8.7; 10.3; 10.4; 12.5<br><br>The amount of BCTs used in the included interventions ranged from 1 to 12, with a mean of 6.2 (SD = 2.96).<br><br>Frequency and Common BCTs:<br>2.2 (n = 23); 1.1 (n = 15); 2.3 (n = 15); 12.5 (n = 15); 1.4 (n = 12); 8.7 (n = 12)	Number of BCTs:<br>Interventions with ≥7 BCTs: SMD = 0.60 (95% CI: 0.18, 1.02), p = 0.005, indicating a significant positive effect on PA.<br>Interventions with <7 BCTs: SMD = 0.18 (95% CI: -0.04, 0.39), p = 0.11, indicating no significant effect.<br><br>The following BCTs were only used in the subgroup with ≥7 BCTs: 1.1, 4.1, 5.1, 5.3, 6.2, 10.9	Methodological Quality (PEDro Scale):<br>13 trials: low risk<br>8 trials: high risk<br><br>Certainty of Evidence (GRADE):<br>Overall quality: low to moderate<br><br>Publication Bias:<br>Funnel plots and Egger's test indicated no significant bias for physical activity outcomes

No	Basic Characteristics (*Author, year, article title, Review category, Number of studies)*	Population AND Sample size	Intervention Delivery mode/technique	PA Intervention effectiveness	BCT labels and frequency of BCTs	Effectiveness of BCTs or components	Evidence quality
**9**	Lin et al., 2022<br><br>Behaviour change techniques that constitute effective planning interventions to improve physical activity and diet behaviour for people with chronic conditions: a systematic review<br><br>SR<br><br>52 studies<br>45 RCTs<br>7 quasi-experimental studies	Community-dwelling adults with at least one chronic condition (e.g., obesity, type 2 diabetes, cardiovascular disease)<br><br>Sample size: NA	Delivery modes:<br>Face-to-face (individual or group sessions)<br>Online/computer-based (web or smartphone app)<br>Mixed formats (combining face-to-face + online)<br><br>Duration: NA	Action planning–based interventions were generally effective in improving PA , especially when combined with additional BCTs that enhance self-efficacy and self-regulation	36 unique BCTs identified across 52 studies:<br>1.1; 1.2; 1.4; 1.5; 1.6; 1.7; 1.8; 1.9; 2.2; 2.3; 2.4; 2.6; 2.7; 3.1; 3.2; 4.1; 5.1; 5.3; 5.6; 6.1; 6.2; 7.1; 8.2; 8.3; 9.1; 9.2; 10.3; 10.4; 10.5; 11.1; 12.1; 12.2; 12.5; 13.2; 15.1; 15.4<br><br>On average, each planning intervention arm used around 8 BCTs in addition to action planning<br><br>Most frequently used BCTs (besides action planning) included: 5.1 (78%); 1.1 (71%); 3.1 (69%); 1.2 (68%); 12.5 (56%); 4.1 (53%); 2.3 (52%); 2.2 (40%)	1.1, 3.1, and 3.2 are specific facilitators of PA planning	RCTs were assessed using ROB: many had unclear/high risk in performance and reporting bias<br><br>Non-RCTs (ROBINS-I tool): Only five trials were rated low risk across all domains

No	Basic Characteristics (*Author, year, article title, Review category, Number of studies)*	Population AND Sample size	Intervention Delivery mode/technique	PA Intervention effectiveness	BCT labels and frequency of BCTs	Effectiveness of BCTs or components	Evidence quality
**10**	Agirre-Elordui et al., 2024<br><br>Physical activity maintenance in colorectal cancer survivors after an exercise intervention applying behaviour change techniques: a systematic review and meta-analysis<br><br>SR + MA<br><br>5 studies<br>All RCTs	Colorectal cancer survivors (CRCS) older than 18 years who completed their primary treatment and were in follow-up care.<br><br>N = 906 (range: 42–347 per study)	Combined PA and BCT. Features included: pedometers, PA diaries, educational materials, motivational interviews, and supervised exercise sessions<br><br>Duration: 3 to 12 months<br><br>Follow-up: 3 to 12 months	MVPA: SMD = 0.22 (95% CI: 0.09, 0.35), $I^2 = 15.4\%$ (follow up: 3-12 months)	Total 22 BCTs Used:<br>1.1; 1.2 ; 1.3 ; 1.4 ; 1.5 ; 2.2 ; 2.3; 3.2; 3.3; 4.1; 5.1; 5.3; 6.1; 7.1; 8.1 8.7; 9.1; 9.2; 12.1; 12.5; 13.2; 15.1<br><br>The interventions included at least 15 [44] of the possible 93 techniques and a maximum of 19 [41] (mean = 17.2 BCT)<br><br>Most used BCTs:<br>1.1(n = 5) ; 1.3(n = 5) ; 1.4(n = 5) ; 1.5(n = 5) ; 2.2(n = 5) ; 2.3(n = 5) ; 4.1(n = 5) ; 5.1(n = 5) ; 7.1(n = 5) ; 8.1(n = 5) ; 9.1(n = 5); 12.5(n = 5); 15.1(n = 5); 3.3 (n = 4);1.2 (n = 3); 6.1 (n = 3); 8.7 (n = 3); 9.2 (n = 3)	Studies with "very promising" results implemented unique BCTs, such as 6.1 and 12.1<br><br>13.2 was applied only in studies categorized as "not promising"	ROB: Concerns included lack of blinding, self-reported outcomes, and selective reporting<br><br>Evidence certainty: Assessed using GRADE guidelines.<br>Overall certainty: Moderate (downgraded due to risk of bias)

No	Basic Characteristics (*Author, year, article title, Review category, Number of studies)*	Population AND Sample size	Intervention Delivery mode/technique	PA Intervention effectiveness	BCT labels and frequency of BCTs	Effectiveness of BCTs or components	Evidence quality
**11**	Cooper et al., 2023<br><br>Behavior change techniques in digital physical activity interventions for breast cancer survivors: a systematic review<br><br>SR<br><br>20 studies<br>13 RCTs<br>7 Quasi-experimental studies	Female breast cancer survivors, aged ≥18 years, currently receiving or having completed treatment<br><br>N = 1,726	Predominantly digital platforms delivering PA behavior change interventions<br><br>Duration: 4 weeks to 12 months<br><br>Follow-up: 3 to 12 months	12 of 20 studies reported significant improvements in PA compared to control groups	Total 27 BCTs Used:<br>1.1; 1.2; 1.4; 1.6; 1.7; 2.1; 2.2; 2.3; 3.1; 3.3; 4.1; 5.1; 6.1; 7.1; 8.1; 8.3; 8.7; 9.1; 9.2; 10.3; 10.4; 10.6; 10.9; 12.1; 13.5; 15.1; 15.4<br><br>Used BCT (mean 4 ± 1, range 2–13)<br><br>Most Frequently Used BCTs:<br>2.3 (n = 17, 85%); 1.1 (n = 15, 79%); 3.1 (n = 11, 58%); 2.2 (n = 10, 50%); 1.2 (n = 8, 42%); 1.4 (n = 8, 42%)	No clear relationship between the number of BCTs used and PA outcomes.<br>Interventions with as few as 2 BCTs achieved significant improvements in PA.<br>Interventions using 2.3 and 1.1 were consistently more effective	Assessed using the Mixed Methods Appraisal Tool (MMAT). Key Issues:<br>Lack of blinding of outcome assessors in RCTs<br>Insufficient confounding control in non-randomized studies<br>Retention rates below 80% in several studies

No	Basic Characteristics (*Author, year, article title, Review category, Number of studies)*	Population AND Sample size	Intervention Delivery mode/technique	PA Intervention effectiveness	BCT labels and frequency of BCTs	Effectiveness of BCTs or components	Evidence quality
**12**	Ester et al., 2021<br><br>Current Evidence and Directions for Future Research in eHealth Physical Activity Interventions for Adults Affected by Cancer: Systematic Review<br><br>SR<br><br>67 studies<br>45 RCTs<br>22 Non-RCTs	Adults aged ≥18 years diagnosed with cancer.<br>Cancer Types: Predominantly breast cancer survivors (38%) and multiple cancer types (34%)<br><br>N = 6,655	eHealth Components: Wearable devices (61%), websites (48%), SMS text messaging (28%), mobile apps (27%), email (22%), and telephone contact (37%)<br>Supervision: Partially supervised (27%) and fully unsupervised (72%)<br><br>Duration: 1 to 52 weeks<br><br>Follow-up (12/57 18%), : 12 to 52 months	Significant Increases: 52% (35/67) of studies reported significant PA increases<br><br>PA Maintenance: 41% (5/12) of studies with follow-up reported PA maintenance<br><br>Decreases: Only 1 study reported a significant decrease in PA	Total 64 BCTs Identified: NA<br><br>The mean number of BCTs used across the studies was 13.5 (SD 5.5), and ranged from 5-42<br><br>Most Common BCTs (>90% of studies):<br>2.3; 9.1; 1.1; 12.5<br>Other Common BCTs (>50% of studies):<br>3.1; 1.5; 2.2; 4.1; 5.1; 8.1; 8.3;1.2; 7.1	BCTs Linked to Increased PA:<br>1.1: Weight = 0.657<br>1.4: Weight = 0.645<br><br>eHealth Components Linked to Increased PA:<br>Websites: Weight = 0.656<br>Mobile Apps: Weight = 0.563<br><br>Number of BCTs:<br>Above Mean (13.5 BCTs): Weight = 0.581<br>Below Mean: Weight = 0.486<br><br>These weight analyses revealed that eHealth interventions with more BCTs were more likely to report significant improvements in PA levels	RCTs: 93% had high bias, mainly due to intervention deviations (84%) and outcome measurement issues (68%)<br>Non-Randomized Studies: 95% had critical bias, predominantly from confounding<br><br>Certainty of Evidence: Overall low due to high bias, incomplete reporting, and significant heterogeneity

No	Basic Characteristics (*Author, year, article title, Review category, Number of studies)*	Population AND Sample size	Intervention Delivery mode/technique	PA Intervention effectiveness	BCT labels and frequency of BCTs	Effectiveness of BCTs or components	Evidence quality
**13**	Finne et al., 2018<br><br>Behavior Change Techniques for Increasing Physical Activity in Cancer Survivors: A Systematic Review and Meta-Analysis of Randomized Controlled Trials<br><br>SR + MA<br><br>30 studies<br>All RCTs	Adults (≥18 years) diagnosed with cancer, post–main cancer treatment (e.g., no active chemo except hormonal therapy)<br>Predominantly breast cancer survivors or mixed cancer types<br><br>N = 4,507	Most interventions were fully home-based (16/30)<br>Some combined home- and facility-based components (10/30); a few used only facilities (4/30)<br><br>Delivery Methods: Telephone calls, printed materials, pedometers, and various behavior change components (e.g., rewards, prompts)<br><br>Duration: 1 week to 12 months	Small but significant positive effect (Hedges' g = 0.28, 95% CI 0.18–0.37, I^2 = 54.29%)<br>Effect sizes were generally higher for self-reported PA (g ≈ 0.316) than for objective measures (g ≈ 0.182)	A total of 37 BCTs were ultimately retained in the final coding:<br>1.1; 1.2; 1.3; 1.4; 1.5; 1.6; 1.8; 1.9; 2.2; 2.3; 3.1; 3.2; 4.1; 5.1; 5.2; 5.3; 5.6; 6.1; 6.2; 7.1; 7.3; 8.1; 8.2; 8.7; 9.1; 9.2; 10.3; 10.4; 10.9; 11.2; 12.1; 12.3; 12.5; 13.2; 13.5; 15.4; 16.2<br><br>The included interventions used an average of 10.44 BCTs (Md=11, SD=4.44), ranging from 1–17<br><br>Most Common BCTs:<br>1.1(n = 27); 3.1(n = 27); 1.2(n = 26); 2.3(n = 26); 4.1(n = 24); 8.1(n = 23); 12.5(n = 21)	BCTs Linked to Larger Effects: 7.1; 7.3; 8.7; 10.3; 10.4<br><br>BCTs Linked to Smaller Effects: 5.1; 5.6; 6.2<br><br>No Effect of Total BCT Count: The total number of BCTs used did not predict effect size.<br>Other Moderators:<br>Interventions based on the Theory of Planned Behavior (TPB) showed smaller effects<br>Home-based interventions were linked to larger effects than purely facility-based ones<br>Neither intervention duration nor methodological quality significantly explained effect-size differences	Global Quality Ratings using Effective Public Health Practice Project (EPHPP): 9 studies rated "strong"; 16 "moderate"; 5 "weak"<br><br>Many studies did not report intervention integrity, blinding, or intention-to-treat analyses<br><br>Publication bias is possible (small studies with large effect sizes)

No	**Basic Characteristics** (*Author, year, article title, Review category, Number of studies)*	**Population AND Sample size**	**Intervention Delivery mode/technique**	**PA Intervention effectiveness**	**BCT labels and frequency of BCTs**	**Effectiveness of BCTs or components**	**Evidence quality**
**14**	Grimmett et al., 2019<br><br>Systematic Review and Meta-Analysis of Maintenance of Physical Activity Behaviour Change in Cancer Survivors<br><br>SR + MA<br><br>27 studies<br>All RCTs	Adults (≥18 years) with any cancer diagnosis. Studies included various cancer types (most often breast cancer)<br><br>N = 5,792	Delivery Modes:<br>Ranged from minimal contact (e.g., printed materials) to more intensive (phone-based support, supervised sessions)<br><br>Duration: a single contact to 10 months<br><br>Follow-up: 3 months to 5 years	Between-Group Meta-Analysis (19 studies):<br>SMD = 0.25, 95% CI: 0.16 to 0.35, $I^2 = 36\%$<br>Mean Difference: 40 minutes/week of MVPA more in intervention vs. control	A total of 31 BCTs (from 14 categories) were coded:<br>1.1; 1.2; 1.3; 1.4; 1.5; 1.6; 1.9; 2.2; 2.3; 2.6; 2.7; 3.1; 3.2; 4.1; 4.2; 5.1; 5.2; 5.3; 5.6; 6.1; 7.1; 8.1; 8.6; 8.7; 9.1; 9.2; 10.4; 11.2; 12.5;13.2; 15.1<br><br>Studies included an average of 10.3 BCTs (range 2– 20)<br><br>Most Frequently Used BCTs:<br>1.1(n = 25); 2.3(n = 22); 4.1(n = 19); 9.1(n = 19); 1.2(n = 18); 3.1(n = 18); 12.5(n = 17); 5.1(n = 16); 1.4(n = 15);8.7 (n = 14)	Notable BCTs in More Promising Interventions:<br>1.4; 8.7; 3.1<br><br>Few Distinctions by BCT between highly successful vs. less successful studies, suggesting other factors (e.g., population characteristics, intervention intensity) strongly influence outcomes<br><br>Context Factors: Younger, well-educated populations tended to show greater improvements with moderate-intensity interventions; older or functionally limited populations often required more intensive or supervised support	ROB: Many studies had high or unclear risk for blinding, attrition bias, and use of self-reported PA measures

No	Basic Characteristics (*Author, year, article title, Review category, Number of studies)*	Population AND Sample size	Intervention Delivery mode/technique	PA Intervention effectiveness	BCT labels and frequency of BCTs	Effectiveness of BCTs or components	Evidence quality
**15**	Hailey et al., 2022<br><br>A systematic review of behaviour change techniques used in interventions to increase physical activity among breast cancer survivors<br><br>SR<br><br>27 studies<br>26 RCTs<br>1 quasi-experimental study	Women diagnosed with breast cancer, having completed main cancer treatment (except possibly ongoing hormonal therapy) in the past 5 years<br><br>N = 3,656	Delivery Context:<br>Home-based Behavior change strategies via activity trackers (e.g., accelerometer, pedometer) with feedback, delivered during or after inpatient care.(n=15), facility/community-based (n=5), or blended (n=7)<br><br>Duration: 8 to 104 weeks<br><br>Follow-up: 12 to 24 weeks	Very Promising (n=11)<br>Quite Promising (n=13)<br>Non-Promising (n=3)	Total 24 BCTs were used at least once:<br>1.1; 1.2; 1.4; 1.5; 2.1; 2.2; 2.3; 2.4; 2.5; 2.6; 3.1; 3.2; 3.3; 4.1; 5.1; 6.1; 6.2; 7.1; 8.1; 9.1; 10.1; 10.4; 12.1; 12.5<br><br>Average six BCTs per study (range 3–9)<br><br>Most Commonly Reported BCTs:<br>4.1 (n=23, 85%)<br>1.1 (n=22, 81%)<br>12.5 (n=13, 48%)<br>2.3 (n=11, 41%)<br>6.1 (n=10, 37%)<br><br>No differences were identified in number of BCTs between promising and non- promising interventions	Promise Ratio Approach:<br><br>12.5 had the highest promise ratio, followed by 1.1 and 2.3<br><br>Theory Considerations:<br>Many promising interventions did not explicitly mention a theoretical framework, though SCT and TTM were common.<br>Some evidence suggests combining TTM + SCT could be particularly effective	Quality assessment：5 studies rated strong/high quality; 17 moderate and 5 low<br><br>ROB Common Biases:<br>Randomization and selection of reported results were the main concerns.<br>Difficulty in blinding participants and staff to PA interventions.<br>High attrition rates in longer studies; not always clearly handled

No	Basic Characteristics (*Author, year, article title, Review category, Number of studies)*	Population AND Sample size	Intervention Delivery mode/technique	PA Intervention effectiveness	BCT labels and frequency of BCTs	Effectiveness of BCTs or components	Evidence quality
**16**	Hallward et al., 2020<br><br>Behaviour change techniques in physical activity interventions for men with prostate cancer: A systematic review<br><br>SR<br><br>15 studies<br>10 RCTs<br>2 quasi-experimental studies<br>3 pre-experimental studies	Men diagnosed with prostate cancer<br><br>N = 2,208	Delivery modes:<br>Clinic-based (supervised) (1 study)<br>Home-based (3 studies)<br>Both clinic and home-based (11 studies)<br>Intervention features:<br>Some interventions included telephone support or booster sessions to enhance adherence<br><br>Duration: 6 weeks to 6 months	Very Promising (n = 6)<br>Unclear promise (n = 5)<br>Non-Promising (n = 4)	21 distinct BCTs appeared in at least one of the 15 interventions.:<br>1.1; 1.2; 1.4; 1.5; 2.2; 2.3; 2.6; 3.1; 3.2; 4.1; 4.2; 5.3; 6.1; 7.1; 8.1; 8.6; 8.7; 9.1; 10.2; 10.3; 12.5<br><br>The studies included a mean 6.87 BCTs (range = 3–10)<br><br>The most commonly used BCTs included:<br>8.1 (n = 15); 4.1 (n = 11); 2.3 (n = 9); 12.5 (n = 9); 3.1 (n = 8); 8.6 (n = 8); 8.7 (n = 7)	Most effective BCTs: Six BCTs were classified as "promising" due to their frequent appearance in "very promising" interventions:<br>3.1; 4.2; 5.3; 7.1; 9.1; 12.5<br><br>Significant BCT: the BCT 4.1 appeared only in promising interventions, suggesting its potential as a key component for success.<br><br>The number of BCTs implemented in promising interventions did not significantly differ from the number of BCTs implemented in non-promising interventions	Quality assessment: 1 high-quality study; 8 moderate-quality studies; 6 low-quality studies<br><br>ROB: 10 studies underpowered for a moderate effect; most studies specified inclusion criteria; several studies lacked full outcome reporting or had selective reporting; randomization and blinding mostly addressed in RCTs; only one study reported full validity and reliability of outcome measures
**17**	Mbous et al., 2020<br><br>A systematic review and meta-analysis of physical activity interventions among colorectal cancer survivors<br><br>SR + MA<br><br>10 studies<br>All RCTs	Adult colorectal cancer survivors (CRCs) at any stage of CRC, pre-, during, or post-treatment<br><br>N = 2,364	Interventions used various delivery methods, including telephone counseling, mailed materials, face-to-face consultations, structured programs, motivational interviewing, smartphone apps, and group meetings. Some studies combined multiple methods, such as face-to-face, telephone, and printed materials<br><br>Duration: NA	9 of 10 studies reported positive improvements in PA<br><br>The meta-analysis (8 RCTs reporting sufficient data) yielded a small but significant overall effect size = 0.26 (95% CI: 0.13, 0.38)	Total BCTs Identified 24 BCTs:<br>1.1; 1.2; 1.3; 1.4; 1.5; 1.6; 1.7; 2.2; 2.3; 2.4; 3.1; 3.2; 3.3; 4.1; 5.1; 5.3; 6.1; 7.1; 8.1; 8.2; 8.6; 8.7; 14.6; 14.8<br><br>The mean BCTs used were 9.9 BCTs (range: 8–17)<br><br>Most commonly used BCTs included:<br>1.1 (n = 10); 1.3 (n = 10); 1.4 (n = 9); 1.2 (n = 9); 2.3 (n = 8); 2.2 (n = 6)<br><br>BCT subgroup analysis revealed that a number of BCTs could positively influence PA behavior	Subgroup analysis (based on BCT presence/absence) indicated that certain techniques were associated with small but positive effect sizes 8.7 emerged with the highest effect size (d = 0.40) among the studied BCTs, suggesting that gradually escalating PA goals can be especially helpful for adherence	GRADE ratings: Post-intervention MVPA was rated moderate quality (downgraded due to risk of bias and moderate heterogeneity); MVPA at follow-up (3–12 months post-intervention) rated high quality (limited heterogeneity)<br><br>ROB: Most frequent concern was lack of blinding (participants and/or outcome assessors) leading to possible detection bias

No	Basic Characteristics (*Author, year, article title, Review category, Number of studies)*	Population AND Sample size	Intervention Delivery mode/technique	PA Intervention effectiveness	BCT labels and frequency of BCTs	Effectiveness of BCTs or components	Evidence quality
**18**	Meyer-Schwickerath C et al., 2022<br><br>Efficacy of face-to-face behavior change counseling interventions on physical activity behavior in cancer survivors – a systematic review and meta-analysis<br><br>SR + MA<br><br>14 studies<br>All RCTs	Adult cancer survivors , age >18 years,<br>N = 1,666	Intervention mode: Exclusively face-to-face behavior change counseling targeting PA adoption or maintenance (e.g., motivational interviewing, educational-based counseling, lifestyle interventions)<br><br>Duration: a single contact to 12 months<br><br>Follow-up (7/14, 50%): 6 weeks to 2 years	SMD = 0.22 (95% CI: 0.11, 0.33)<br><br>Five studies observed sustained improvements in PA at follow-up (ranging 6 weeks to 12 or 24 months)	Across all interventions, 24 unique BCTs were identified:<br>1.1; 1.2; 1.3; 1.4; 1.5; 1.7; 2.2; 2.3; 2.7; 3.1; 4.1; 5.1; 6.1; 7.1; 8.1; 8.4; 8.7; 9.1; 9.2; 10.9; 11.2; 12.5; 13.2<br><br>Studies included an average of 8.2 BCTs (range 2- 15)<br><br>The most frequently reported BCTs (≥10 studies) were:<br>2.3 (n = 13); 1.1 (n = 11); 4.1 (n = 11); 2.2 (n = 11); 3.1 (n = 10); 12.5 (n = 8); 1.2 (n = 7); 6.1 (n = 7) ;8.7 (n = 7)<br><br>There is no difference in the amount of BCTs implemented between interventions with effect sizes above 0.25 and interventions with effect sizes 0.10.	Overall, no single BCT or combination was universally linked to the largest effects<br><br>However, the BCTs such as 8.7, 1.4, 8.4, and 9.1 appeared more often in interventions with higher effect sizes (>0.25) and not in those with near-zero effect<br><br>8.7 emerged as a particularly promising approach (participants gradually increase activity difficulty)	ROB: 0 studies underpowered; most studies specified inclusion criteria; several lacked full outcome reporting; randomization and blinding mostly addressed; only one study reported full validity and reliability<br><br>GRADE assessment: Overall low quality of evidence, downgraded for risk of bias (lack of concealment, no blinding) and imprecision (underpowered studies)

Notes: BCT(s) = behavior change technique(s); BCTTv1 = Behavior Change Technique Taxonomy version 1; BMI = body mass index; CHD = coronary heart disease; CI = confidence interval; CRCS/CRCs = colorectal cancer survivor(s); CVD = cardiovascular disease; DBCI(s) = digital behavior change intervention(s); EPHPP = Effective Public Health Practice Project quality assessment tool; GRADE = Grading of Recommendations, Assessment, Development and Evaluation; IQR = interquartile range; LPA = light physical activity; MA = meta-analysis; Md = median; MMAT = Mixed Methods Appraisal Tool; MVPA = moderate-to-vigorous physical activity; Non-RCT = non-randomized controlled trial; OA = osteoarthritis; PA = physical activity; PEDro scale = Physiotherapy Evidence Database scale; PMP/PMSK = persistent musculoskeletal pain; RCT = randomized controlled trial; ROB = risk of bias; ROBINS-I = Risk Of Bias In Non-randomized Studies of Interventions; SCT = Social Cognitive Theory; SD = standard deviation; SMD = standardized mean difference; SR = systematic review; TPB = Theory of Planned Behavior; TTM = Transtheoretical Model.
